# The Role of Amino Acid Permeases and Tryptophan Biosynthesis in *Cryptococcus neoformans* Survival

**DOI:** 10.1371/journal.pone.0132369

**Published:** 2015-07-10

**Authors:** João Daniel Santos Fernandes, Kevin Martho, Veridiana Tofik, Marcelo A. Vallim, Renata C. Pascon

**Affiliations:** 1 Departamento de Ciências Biológicas, Instituto de Ciências Ambientais, Químicas e Farmacêuticas, Universidade Federal de São Paulo, Campus Diadema, Laboratório de Interações Microbianas (Laboratory 29), Rua Arthur Ridel, 275, 09972–270, Bairro Eldorado, Diadema, SP, Brazil; 2 Universidade de São Paulo, Avenida Prof. Lineu Prestes, 2415 Edifício ICB – III, Cidade Universitária, CEP 05508–900, São Paulo, SP, Brazil; Yonsei University, REPUBLIC OF KOREA

## Abstract

Metabolic diversity is an important factor during microbial adaptation to different environments. Among metabolic processes, amino acid biosynthesis has been demonstrated to be relevant for survival for many microbial pathogens, whereas the association between pathogenesis and amino acid uptake and recycling are less well-established. *Cryptococcus neoformans* is an opportunistic fungal pathogen with many habitats. As a result, it faces frequent metabolic shifts and challenges during its life cycle. Here we studied the *C*. *neoformans* tryptophan biosynthetic pathway and found that the pathway is essential. RNAi indicated that interruptions in the biosynthetic pathway render strains inviable. However, auxotroph complementation can be partially achieved by tryptophan uptake when a non preferred nitrogen source and lower growth temperature are applied, suggesting that amino acid permeases may be the target of nitrogen catabolism repression (NCR). We used bioinformatics to search for amino acid permeases in the *C*. *neoformans* and found eight potential global permeases (*AAP*1 to *AAP*8). The transcriptional profile of them revealed that they are subjected to regulatory mechanisms which are known to respond to nutritional status in other fungi, such as (i) quality of nitrogen (Nitrogen Catabolism Repression, NCR) and carbon sources (Carbon Catabolism Repression, CCR), (ii) amino acid availability in the extracellular environment (SPS-sensing) and (iii) nutritional deprivation (Global Amino Acid Control, GAAC). This study shows that *C*. *neoformans* has fewer amino acid permeases than other model yeasts, and that these proteins may be subjected to complex regulatory mechanisms. Our data suggest that the *C*. *neoformans* tryptophan biosynthetic pathway is an excellent pharmacological target. Furthermore, inhibitors of this pathway cause *Cryptococcus* growth arrest *in vitro*.

## Introduction

Many microbial pathogens are subjected to rapidly changing environmental conditions and require robust metabolic strategies to survive and succeed during these changes [1–3]. Yeasts from the *Cryptococcus* genus—mainly *C*. *neoformans* (var. *neoformans* and var. *grubii*) and *C*. *gattii* species—face this type of challenge during their life cycle [4]. Both species can be found in vegetables, fruits, trees, soil, decaying wood, and bird guano. They are adapted to the animal and human environment, encountering extreme nutritional and physical-chemical alterations when entering the host [5–7]. *C*. *gattii* is associated with eucalyptus trees and other plant niches, can cause disease in immunocompetent patients, and is responsible for recent meningoencephalitis outbreaks in North America [8, 9]. In contrast, *C*. *neoformans* (var. *neoformans* and var. *grubii*) is the most clinically prevalent species, and is the main cause of fungal meningitis in immune suppressed populations, especially patients with HIV/AIDS [5, 10, 11]. In 2009, Park and collaborators evaluated the global burden of cryptococcal meningitis based on the population studied as part of the UN’s 2007 Programme on HIV/AIDS and found that there is an estimated 957,900 new cases per year worldwide (and 624,700 deaths), most of which are in sub-Saharan Africa [12].

Cryptococcosis is considered an invasive fungal infection (IFI) that is difficult to treat, due to the relatively few available antifungal [5, 10, 13]. Many virulence factors related to infection and disease have been described over the years, such as: capsule, melanin, phospholipase, urease, thermotolerance, and multi-stress response [14–16]. Regulatory circuits have been described in *S*. *cerevisiae* and other model fungi that control the assimilation of nutrients such as carbon, nitrogen, phosphate, purines, pyrimidines and amino acids [17]. Nutrient acquisition mechanisms have also been recently described in *C*. *neoformans*, often in association with virulence. Nutrient sensing, transport, biosynthesis, storage and recycling have been reported as key factors for survival in the host cell [44]. The acquisition of carbon, nitrogen, phosphate, iron, pyrimidines and amino acids has been directly implicated in the expression of virulence factors as well as in *in vitro* and *in vivo* survival [18–32].

In addition to uptake, amino acid biosynthetic pathways have been reported as key factors for *C*. *neoformans* survival in the host. Several of these genes are essential for growth, and others, when mutated, result in specific auxotrophies and reduced virulence, which makes them interesting pharmacological targets. Nine of the amino acid biosynthetic pathways are absent in humans, suggesting the potential for selective inhibition. Gene deletion in some of these amino acid biosynthetic pathways (lysine, isoleucine, valine, threonine, and methionine) leads to defects in *C*. *neoformans* virulence factors, impaired multi-stress tolerance, hypersensitivity to serum, and increased susceptibility to antifungal drugs; also, all mutants tested so far display virulence attenuation in animal models [27–32]. The threonine biosynthetic pathway, in particular, contains essential genes, since targeted mutations in this pathway have not been successful. This suggests that threonine uptake may not be enough to satisfy resulting auxotrophies, perhaps due to permease inefficiency [27].

Perturbations in aromatic amino acid biosynthesis, mainly tryptophan, affect optimal growth in *Mycobacterium tuberculosis* and *M*. *bovis BCG*. Mutants in these pathways have attenuated virulence or are avirulent, making them strong potential pharmacological targets [33–37]. Shen *et al*. (2009) identified a nitrogenous compound (ATB107) with inhibitory effects on IGPS (3-indol-glycerol phosphate synthase) which is encoded by the *TRP3* gene in *M*. *tuberculosis*. ATB107 has low toxicity in macrophages and inhibits most multi-drug resistant TB strains [36, 37]. Various other compounds have been described over the years as inhibitors of tryptophan biosynthetic enzymes [38–43]. The tryptophan biosynthetic pathway is also the target of anti-metabolites in various organisms, from *Pseudomonas putida*, *Legionella pneumophila* to *Coprinus cinereus* and *S*. *cerevisiae*. However, these compounds have never been tested in *C*. *neoformans* [44–48].

The block in aromatic amino acid biosynthesis in *C*. *neoformans* by sub inhibitory concentrations of glyphosate delays melanization *in vitro* and *in vivo*, leading to prolonged mice survival during experimental *C*. *neoformans* infection [49]. This suggests that tryptophan, tyrosine and phenylalanine biosynthetic pathways may be valid novel drug target candidates; however, tryptophan is the only one with no alternative biosynthetic route.

The mechanisms of amino acid acquisition by membrane bound permeases and recycling have not been explored in *C*. *neoformans*. However, this subject has been extensively explored in *S*. *cerevisiae* [50–56]. In order to expand our knowledge on the nutritional attributes necessary to guarantee survival *in vitro* and *in vivo* and explore new potential pharmacological targets, we studied the tryptophan biosynthetic pathways in *C*. *neoformans*. In this work, we identified and described the four gene products in this pathway: *TRP2* (Anthranilate synthase component I – AS COI), *TRP3* (Anthranilate synthase component II – AS COII), a trifunctional protein, *TRP4* (Anthranilate Phosphoribosyl transferase—APRT) and *TRP5* (tryptophan synthase—TS). Our data highlight several important issues: first, tryptophan biosynthetic pathway is essential in *C*. *neoformans*; second, transcript levels of amino acid permeases vary in response to nutrient source availability, but do not appear sufficient to support tryptophan auxotrophies; lastly, commercial inhibitors of AS COII and TS were tested and are active against both *C*. *neoformans* and *C*. *gattii*, demonstrating the potential application of inhibitors directed at the tryptophan biosynthetic pathway.

## Materials and Methods

### Strains and reagents

*E*. *coli* and *C*. *neoformans* strains used in this work are described in S1 Table. Standard yeast extract-peptone-glucose (YEPD) plates were prepared as 1% yeast extract, 2% peptone, 2% dextrose and 2% agar; Synthetic medium was made with 6.7 g/liter Sigma Yeast Nitrogen Base with or without amino acids and ammonium sulfate, added with the appropriate carbon source (2% dextrose or galactose), nitrogen source (Ammonium sulfate or proline) and 2% agar. Tryptophan (kept in the dark) and uracil were added at 20 mg/L and 10 μg/mL final concentrations, respectively. Hygromycin and G418 were used as selection agents added to the medium at 200 μg/mL.

### Bioinformatics

Tryptophan biosynthetic (*TRP*) and permease genes (*APC*) were identified in *C*. *neoformans* var. *grubii* and *C*. *neoformans* var. *neoformans* genome by BLAST search at Broad Institute (http://www.broadinstitute.org/) and NCBI (http://blast.ncbi.nlm.nih.gov/Blast.cgi). Amino acid sequences from *S*. *cerevisiae TRP* and *APC* genes were used as query (S2 Table). All sequence alignments and phylogenetic trees were made using Lasergene software (DNAStar).

### Gene deletion

The coding region from start to stop codon was replaced by Geneticin or Hygromicin resistance cassettes containing the *Neo* ^R^ or *Hph* ^R^ genes under the control of actin promoter and *Trp*1 terminator. *In vitro* construction of the *trp*Δ::*DRM* alleles was conducted according to published protocols [57, 58] and introduced into *C*. *neoformans* H99 strain by biolistic transformation [59]. Gene deletion constructs were made for all four tryptophan biosynthetic genes (*TRP2*, 3, 4 and 5). S3 Table describes all the primers used in the deletion constructs and S4 Table describes gene deletions, the selectable markers used for each one, and their respective serotypes.

### RNAi

Gene silencing experiments were performed according to the previously published protocol [60]. In brief, two different regions of the coding sequence of *TRP3*—*trp3*.1i (235 bp) and *trp3*.2i (408 bp) and *TRP5*: *trp5*.1i (181 bp) and *trp5*.2i (353 bp)—were PCR amplified. The primers were designed to introduce *Spe*I restriction sites at the 5´and 3´ends of all the amplicons in order to allow cloning at the *Spe*I restriction site of the pIBB103 vector digested with *Spe*I. The linear and dephosphorylated vector was ligated to the PCR-amplified fragments and introduced into *E*. *coli* (HB 101), generating the plasmids described in S5 Table. All plasmids were digested with I-*Sce*I restriction enzyme and purified with QiaQuick prior to biolistic transformation. All transformants were selected on YEPD plates containing 200 μg/mL Geneticin (G418) and 1M sorbitol. After 72 hours at 30°C, 50 colonies obtained by transformation with each plasmid were purified by two rounds of growth in YEPD+G418. A phenotypic screen was created by replica-plating 50 transformants in YEPD+G418, YEPG+G418, SD+G418+uracil, SG+G418+uracil, SD+G418+uracil+tryptophan, and SG+G418+uracil+tryptophan. All plates were incubated at 30 and 25°C. Uracil was added to all synthetic medium compositions since *URA*5 is the sentinel gene in the pIBB103 vector. The influence of nitrogen sources was evaluated by substituting ammonium sulfate by proline [29] at 30 and 25°C. *TRP3* and *TRP5* silencing was confirmed by qPCR.

### Inhibitor sensitivity assay

All anti-metabolites directed to the tryptophan biosynthetic pathway were incorporated to solid medium on petri dishes. Anti-metabolites: 5-fluoroanthranilic acid (5-FAA), 3-hydroxianthranilic acid (3-HAA) and 5-methylanthranilic acid (5-MAA) were purchased from Sigma and stock solutions were prepared as 100 mg/mL in 10% ethanol and used at 5 μg/mL final concentrations. *C*. *neoformans* strains were grown on YEPD medium overnight at 30°C, washed twice in sterile ultrapure water, serial diluted (2x10^6^ to 2x10^2^ cell/mL) and 5 μL of each dilution were spotted on dry plates containing YEPD or SD medium and anti-metabolites [48].

*TRP3* (6-diazo-5-oxo-L-norleucine) and *TRP5* inhibitors [N-(3-Indolylacetyl)-DL-aspartic acid, N-(3-Indolylacetyl)-L-alanine, N-(3-Indolylacetyl)-L-valine) were purchased from Sigma, diluted in sterile water and tested by minimum inhibitory concentration assay according to the CLSI (Clinical and Laboratory Standard Institute). In brief, OPAS1 M27-A2 was applied with slight modifications: microdilution test were made in 96 well microtiter plates in a total volume of 100 μL/well. The cultures were diluted and adjusted to the final concentration of 1–2 × 10^2^ CFU/well in the appropriate media (YEPD or YNB + Glucose + (NH_4_)_2_SO_4_ + tryptophan or YNB + Glucose + Proline + Tryptophan). Viability and count tests were performed by plating 100 μL of cells on YEPD. Two-fold serial dilutions of 6-diazo-5-oxo-L-norleucine (DON) and TRP5 inhibitors N-(3-Indolylacetyl)-DL-aspartic acid (IAAA), N-(3-Indolylacetyl)-L-alanine (IAA) and N-(3-Indolylacetyl)-L-valine (IAV) were tested. Sterilization control containing solely medium was included as negative control. The microtiter plates were incubated at 30°C for 48 h. Growth was determined by reading the absorbance at 530 nm in a plate reader (Epoch, BioTek Instruments), and the minimum inhibitory concentration was considered as the lowest concentration at which at least 90% of growth was inhibited. All tests were performed in biological triplicates. The concentration range tested for each agent was as follows: 500 to 0,06 μM of (DON) and 50 to 0,39 mM (IAAA, IAA, IAV) with 2 fold dilutions.

### Transcriptional analysis by real time PCR

Total RNA for transcriptional pattern analysis was obtained from cells incubated overnight in liquid YEPD under 150 rpm agitation. Cells were harvested, washed twice in sterile ultrapure water and resuspended in the appropriate medium. In the case of RNAi, induction was performed in YEPG and repression in YEPD for 4 hours under selection. RNA extraction was conducted according to the previously published protocol [60]. In brief, 3x10^8^ cells grown under the desired condition were centrifuged and resuspended in 750 μL Trizol (Invitrogen) and the same volume of glass beads. The mixture was homogenized 10 times in vortex for 1 minute intercalated for 2 minutes on ice. The tubes were left to settle at room temperature to allow the glass beads to precipitate. The supernatant was transferred to a clean tube and Trizol extraction was conducted according to the manufacturer’s instructions. RNA was diluted in DEPC-treated water and stored in the freezer in separate aliquots. cDNA synthesis was performed with the RevertAid H minus First Strand cDNA synthesis kit (Thermo Scientific) with Oligo dT and random hexamer primers from 5 μg of total RNA. Real time PCR amplifications were made from diluted templates (1:10) with 800 nM target primers, 300 nM *GPDH*1 (Glyceraldehyde-3-phosphate dehydrogenase) internal control primers, and 1X Power SYBR Green master mix (Life Technologies). Quantification of the transcript levels was performed using the ΔΔ^CT^ method normalizing against *GPDH*1, as previously described [61]. An analysis of variance was performed by Tukey´s multiple comparison test using GraphPad Prism 5.0 software, and *p* values lower than 0.05 were considered statistically significant.

## Results

### Genome organization of the tryptophan biosynthetic genes in *C*. *neoformans*

**Fig 1:**
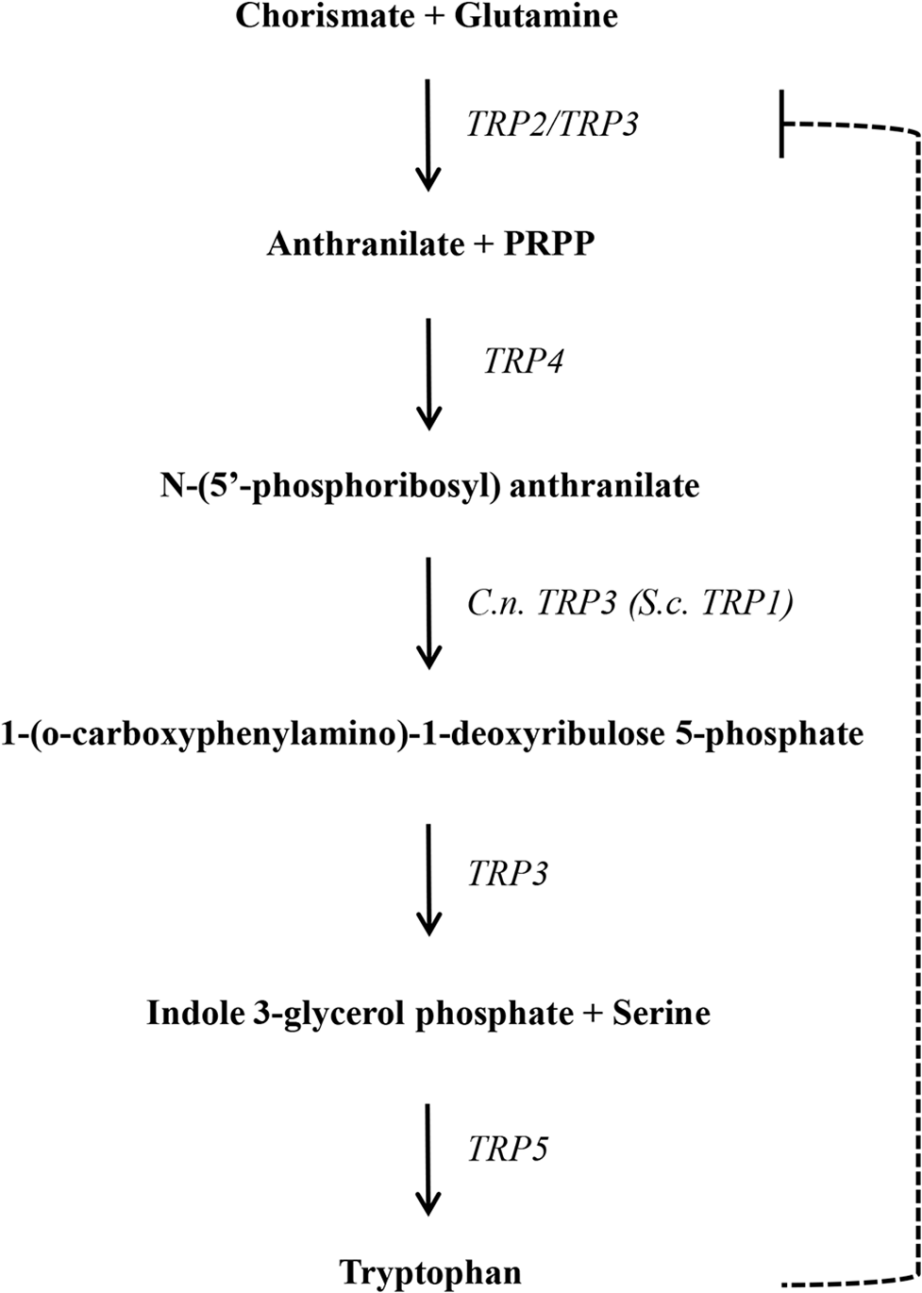
Tryptophan biosynthetic pathway in *C*. *neoformans* and *S*. *cerevisiae*. Genes are depicted on the right side. The dashed line indicates feedback regulation by the end product.

The conversion of chorismate into tryptophan depends on the catalytic steps depicted in Fig 1. In *S*. *cerevisiae*, five genes (*TRP1*, 2, 3, 4 and 5) encode the enzymes involved in this biosynthetic pathway. The amino acid sequence of these proteins were used as queries to search for homologues in *C*. *neoformans* and *C*. *gattii* genomes deposited at the Broad Institute and NCBI to gain knowledge about genome organization of this pathway in *Cryptococcus*. Table 1 shows the *locus* code, enzymatic function, gene and protein sizes and % amino acid similarity of the homologues in *C*. *neoformans* (H99, serotype A and JEC21, serotype D) and *C*. *gattii* (serotype B, strains WM276 and R265). There is a high percentage of similarity among strains of the same species (Table 1).

**Table 1:** Summary of genome organization of the *TRP* genes in *C*. *neoformans* (strains H99 and JEC21) and *C*. *gattii* (strains WM276 and R265).

Gene (catalytic activity)	Strain	Locus Tag	Gene (nt)	Amino Acids	% amino acid similarity*
*TRP*2 (ASCO I—chorismate aminase)	H99	CNAG_06679.2	1869	523	97.9
	JEC21	CNF 03410	1928	534	-
	WM276	CGB_F2090W	1882	534	98.5
	R265	CNBG_1889	1882	534	-
*TRP*3 (ASCO II—Glutamine amidotransferase/Phosphoribosyl-anthranilate isomerase/Indoleglycerol phosphate synthase)	H99	CNAG_04501.2	2396	752	97.5
	JEC21	CNI 00560	2449	752	-
	WM276	CGB_H1160W	2366	752	97.2
	R265	CNBG_2567.2	2526	651	-
*TRP*4 (Anthranilate phosphoribosyl transferase)	H99	CNAG_00811.2	1571	434	97.2
	JEC21	CNA 07880	1732	434	-
	WM276	CGB_A9290C	1521	434	99.5
	R265	CNBG_1124.2	1612	434	-
*TRP*5 (Tryptophan synthase)	H99	CNAG_00649.2	2645	715	96.8
	JEC21	CNA 06290	2623	715	-
	WM276	CGB_A7080C	2545	715	100.0
	R265	CNBG_1280.2	2540	718	-

* % similarity between the two strains of the same species: *C*. *neoformans* (H99 vs. JEC21) and *C*. *gattii* (WM276 vs. R265).

The *in silico* analysis showed that in *S*. *cerevisiae*, Phosphoribosyl-anthranilate isomerase (PRAI) is encoded by a single gene (*TRP*1); in *Cryptococcus*, the conserved domain corresponding to this enzymatic function seems to be encoded by the *TRP3* gene, according to the PROSITE (http://prosite.expasy.org) and Motif Scan databases (http://myhits.isb-sib.ch/cgi-bin/motif_scan), in addition to its usual function. This configuration would produce a triple function protein. This compact arrangement produces an enzyme that acts in the first, third and fourth steps of the pathway (Fig 1). *Cryptococcus* tryptophan synthase encoded by the *TRP5* gene has the same configuration as *S*. *cerevisiae* and other fungi, that is, the A and B subunits are encoded by the same gene, and both enzymatic functions are confined to the same protein. The remaining enzymes encoded by *TRP*2 (chorismate aminase) and *TRP*4 (Anthranilate phosphoribosyl transferase) have the same organization in *S*. *cerevisiae* and *C*. *neoformans*, according to the *in silico* analysis.

The comparison of *TRP* proteins with different Ascomycetes and Basiomycetes showed that the closest similarities can be found between *C*. *neoformans* and *C*. *cinerea*, followed by *U*. *maydis*.

Regarding *TRP3*, the bioinformatic approach detected that other Ascomycetes and Basiomycetes also encode trifunctional proteins (*Ustilago maydis*, *Coprinopsis cinerea*, *Aspergillus fumigatus*, *Ajellomyces dermatitidis*, *Coccidioides immitis* and *Paracoccidioides brasiliensis*). Braus suggests that the separation of *TRP*1 from *TRP3* is a recent event in evolution and that the most ancient and widespread configuration is a trifunctional organization [62].

The *in silico* analysis suggested that *C*. *neoformans* and *C*. *gattii* encode the necessary enzymatic apparatus to synthesize tryptophan. However, biochemical data are necessary to characterize enzymatic properties.

### TRP biosynthetic pathway is the target of anti-metabolite inhibitors

**Fig 2:**
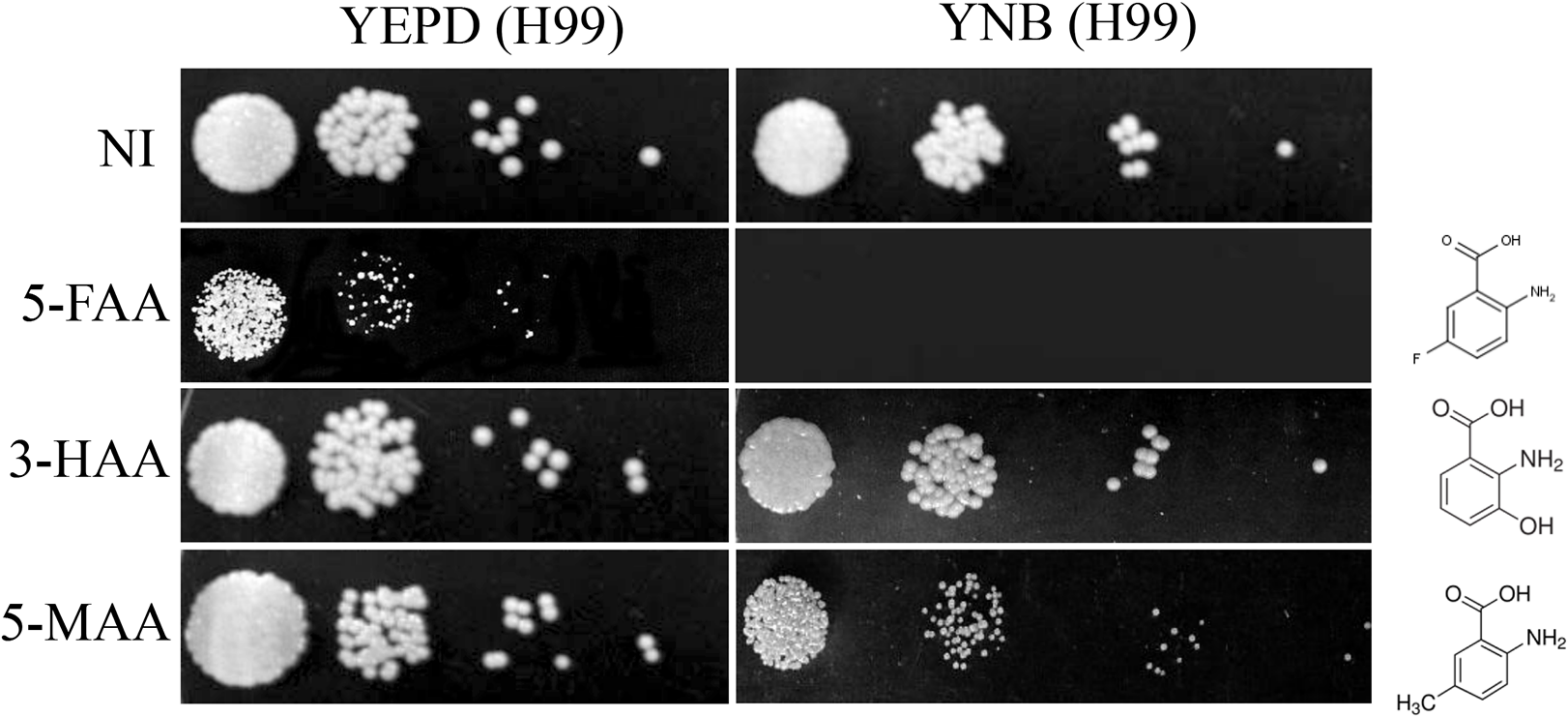
Effect of anti-metabolites in *C*. *neoformans*. Serial dilutions of the H99 strain were spotted in rich (YEPD) and synthetic medium (YNB supplemented with ammonium sulfate and glucose) containing 5μg/mL of anti-metabolite. 5-FAA = 5-fluoroanthranilic acid; 3-HAA = 3-hydroxianthranilic acid; 5-MAA = 5-methylanthranilic acid and NI = no inhibitor.

The tryptophan biosynthetic pathway may be a valuable candidate for drug targets since its end product is one of the nine essential amino acids in animal cells; moreover, it has no alternative biosynthetic routes in plants, microorganisms or api-complexan parasites. Once the genes that act in this pathway were identified *in silico*, we expected that a functional pathway should be the target of anti-metabolites, which should lead to growth arrest in the wild type. Also, one can predict that interruptions in the pathway caused by mutations on the *TRP* genes would confer resistance to this type of growth inhibitor. Fig 2 shows the effects of 5-fluoroanthranilic acid (5-FAA), 3-hydroxianthranilic acid (3-HAA) and 5-methylanthranilic acid (5-MAA) on the growth of the H99 strain in two different conditions: rich and synthetic medium. All three anti-metabolites serve as substrate for Anthranilate phosphoribosyl transferase (second step in the pathway, Fig 1). They act by generating toxic tryptophan analogues, which, once incorporated into proteins, disturb growth. 5-FAA in synthetic medium supplemented with exogenous tryptophan completely inhibited growth, demonstrating that the biosynthetic pathway is functional. The other two inhibitors did not have any effect on growth, likely because they may not be metabolized as well as 5-FAA [45, 48]. This result motivated us to investigate the effects of *TRP* gene deletion on *C*. *neoformans* survival and virulence.

### Deletion of the *TRP* genes

Deletion constructs bearing *Neo* ^R^ (*TRP2* and *TRP4*) and *Hph* ^R^ (*TRP3* and *TRP5*) resistance markers were assembled to individually knock out each *TRP* gene (S4 Table). In all cases, the deleted region comprised the entire coding sequence, from start to stop codons. Deletion cassettes were introduced in the H99 strain by biolistic transformation and selections were conducted in YEPD, added with the appropriate antibiotic (G418 or hygromycin). Phenotypic screens for tryptophan auxotrophies were conducted in synthetic medium (YNB) supplemented with ammonia sulfate as the sole nitrogen source, with or without tryptophan. A total of 2376 transformants were analyzed and no auxotrophies were identified (S6 Table), suggesting TRP genes are recalcitrant to deletion. To establish the causes of this refractory behavior, we confirmed the frequency of homologous integration in a distinct locus by including an *ura*4Δ::*Neo* ^R^ deletion construct in our biolistic experiments [22]. We chose the *ura*4 control because of its easy phenotypic screen (uracil auxotrophy) for homologous integration. Among 50 transformants resistant to G418, 22% were auxotrophic for uracil, showing that, at least for this locus, the frequency of homologous integration is high enough to find auxotroph mutants. Therefore, the lack of tryptophan mutants is likely unrelated to the biolistic process itself. Also, since the four TRP genes are located in different regions of the genome, it is unlikely that this refractory deletion characteristic is related to the chromosomal topology. We produced a *trp5*Δ::*Hph* ^R^ construct for *C*. *neoformans* serotype D and introduced it in the JEC21 strain. Among 150 transformants, no auxotroph mutant was found, suggesting that whatever the reason for the lack of tryptophan auxotroph, it is shared by serotype A and D.

Also, assuming the hypothesis that *trp* mutants have slower growth and that this phenotype could result in difficult selection, we selected the transformants in YNB supplemented with ammonium sulfate, amino acids and 5-FAA; this condition should allow only the growth of tryptophan mutants [48], since we showed that the wild type H99 strain is sensitive to this anti-metabolite (Fig 2). The results show that among 571 transformants resistant to 5-FAA, none were tryptophan auxotrophs (S6 Table). In order to promote a more stringent selection, we used the same anti-metabolite strategy but exchanged the nitrogen source from ammonium sulfate to proline, which should alleviate nitrogen catabolite repression (NCR) and increase amino acid uptake. However, we still did not identify any auxotrophic mutants among the 150 transformants screened. 5-FAA has been used to select spontaneous *trp* ^-^ mutants in *S*. *cerevisiae* [48, 63]. According to Toyn *et al*. (2000), 10% of these mutants are resistant to the anti-metabolite but are not tryptophan auxotrophs. This phenotype may be associated with mutations that affect inhibitor uptake or low incorporation of 5-fluorotryptophan during protein synthesis. However, that remains to be demonstrated in *S*. *cerevisiae* and *C*. *neoformans*.

Previous work showed that *TRP3* is a single copy gene [64]. Our bioinformatic analysis supported this observation, and southern blots for the *TRP* genes further confirmed this (data not shown). Our data suggest that *TRP* genes may be essential in *C*. *neoformans*.

### *TRP3* and *TRP5* are essential genes in *C*. *neoformans*

**Fig 3:**
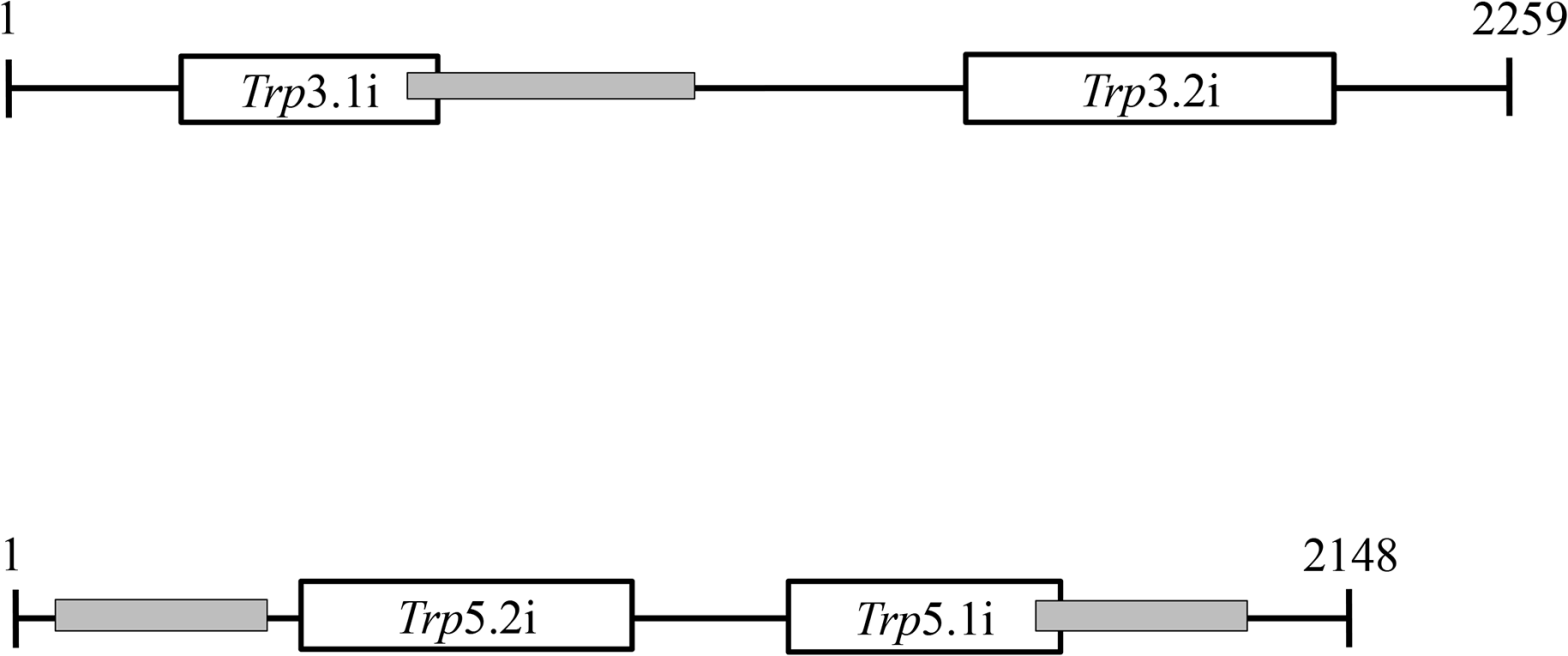
Position of the fragments used to induce RNA interference in *TRP3* and *TRP5*. The numbers and black lines represent the coding region of *TRP*3 and *TRP*5. The position and length of RNAi fragments in the coding region are represented as open boxes. *trp*3.1i and *trp*5.1i fragments are smaller (280 pb and 233 pb, respectively) than *trp*3.2i and *trp*5.2i fragments (402 pb and 353 pb, respectively). Gray boxes indicate the position of the fragments amplified in qPCR.

In order to test the hypothesis that the tryptophan biosynthetic pathway is essential in *C*. *neoformans*, we used a combination of galactose inducible promoter and RNAi approaches to control the expression of *TRP3* and *TRP5* genes. This experiment was conducted in *C*. *neoformans* serotype D strain JEC21, since RNA silencing does not operate efficiently in serotype A [60]. Two different regions of each gene (Fig 3) were expressed under the control of two convergent *Gal*7 promoters and terminators, according to the system developed by [65] and described in detail elsewhere [60]. The smaller interfering region presented in Fig 3 had the best results for both *TRP3* and *TRP5*. Previous report indicated that shorter interfering regions provide the best RNA silencing effect [60]. A total of 50 transformants for each gene (*trp*3.1i and *trp*5.1i) plus the negative control (empty pIBB103) were randomly selected in YEPD + G418. After two rounds of purification in YEPD + G418 (RNAi repressive medium), the transformants were tested in YEPG + G418 (RNAi inducible medium) plus and minus tryptophan. All 50 transformants containing the empty pIBB103 grew on repressive and inducible media (YEPD and YEPG, respectively). However, out of 50 transformants analyzed for each gene, 18 (*trp3*.1i) and 21 (*trp5*.1i) transformants grew only in the RNAi repressive condition (Dextrose). No growth was observed for the 39 transformants in the inducible condition, regardless of tryptophan supplementation. The remaining transformants of each type grew in all four conditions, indicating partial or no interference, which is typical of RNAi phenomena [60].

**Fig 4:**
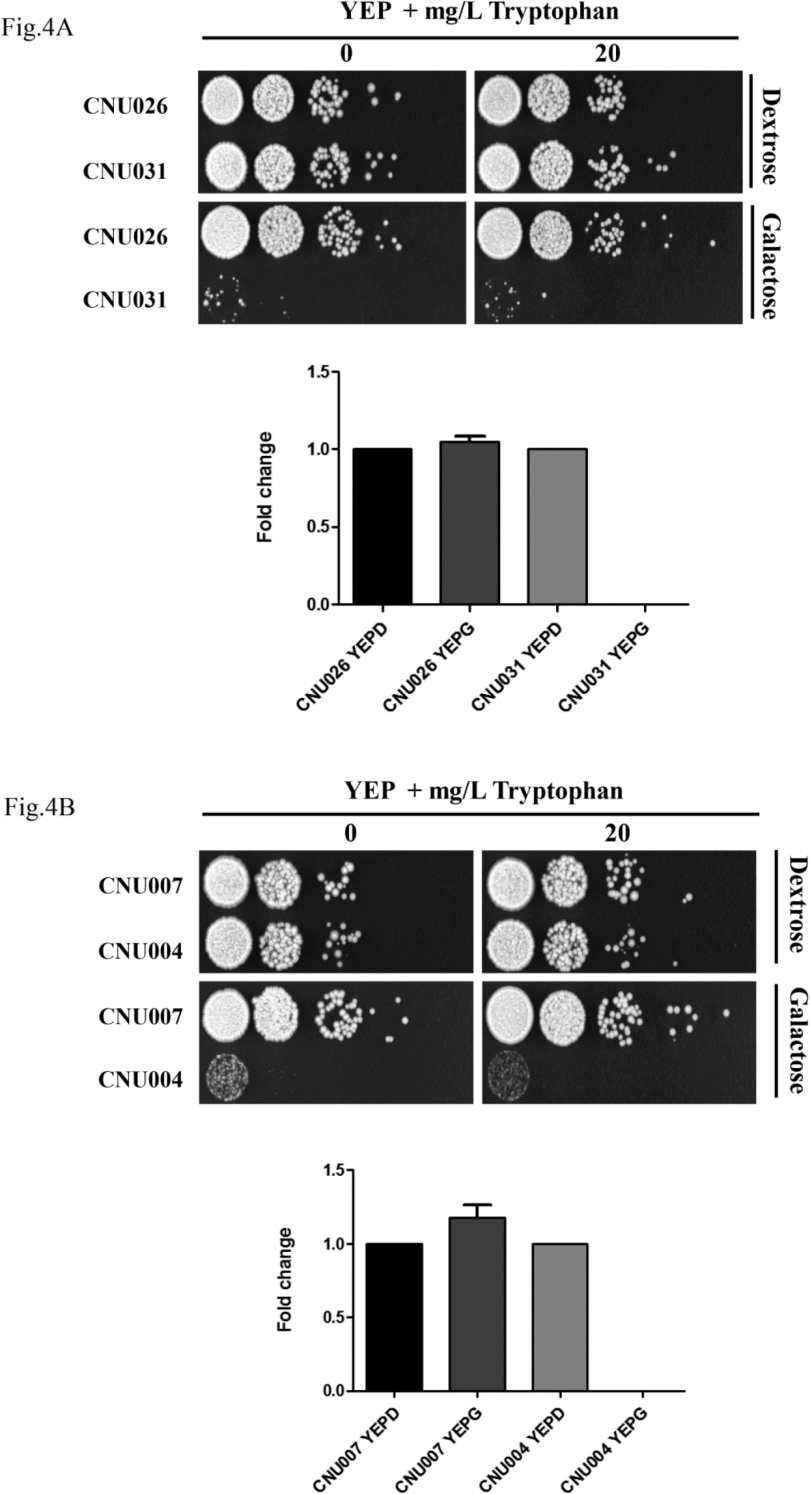
(A) *TRP3* and (B) *TRP5* RNA interference. Ten-fold serial dilutions (10^4^ to 1 cell) were spotted in YEP medium with dextrose (RNAi repressing condition) or galactose (RNAi inducing condition), with or without 20 mg/L tryptophan supplementation. CNU026 and CNU007 are control strains (empty pIBB103 plasmids) and CNU031 and CNU004 are test strains (*trp*3.1i and *trp*5.1i in pIBB103 plasmids). The graphs in Fig 4A and 4B show the relative quantification (RQ) of *TRP3* and *TRP5* transcripts in RNAi inducing (YEPG, galactose) and repressing (YEPD, dextrose) conditions for control strains (CNU026 and CNU007) and mutant strains (CNU031 and CNU004). The RNAi mutants CNU031 and CNU004 are representative of 39 mutants obtained in a screen comprising 100 transformants.

Three mutants for each gene were analyzed in more detail and submitted to qPCR for transcript quantification. One mutant for each *trp*3.1i (CNU031) and *trp*5.1i gene (CNU004) and two controls (empty pIBB103, CNU026 and CNU007) were chosen to illustrate the growth arrest in the presence of galactose, regardless of tryptophan supplementation (Fig 4A and 4B). In order to prove that the phenotype is associated with specific RNA silencing, the abundance of *TRP3* and *TRP5* transcripts were evaluated. All four strains showed in Fig 4 were grown for four hours in liquid rich medium (YEP) containing dextrose or galactose. The total RNA from these cultures was extracted and the expression profiles of the controls (CNU026 and CNU007), *TRP3* (CNU031) and *TRP5* (CNU004) in the two conditions were detected by qPCR and normalized by *GPDH*. The graphs in Fig 4A and 4B show that the expression level of *TRP3* and *TRP5* is stable between the repressive and induced conditions regarding the controls (CNU026 and CNU007). However, *TRP3* and *TRP5* transcripts were below detection levels in CNU031 (Fig 4A) and CNU004 (Fig 4B) under the RNAi induced condition (YEPG), indicating that the phenotypes correlate with transcript levels and, therefore, that RNA silencing was successfully induced by galactose. These results strongly suggest that *TRP3* and *TRP5* are essential genes in serotype D.

Considering the refractory gene deletion phenomena encountered for all four *TRP* genes in serotype A and *TRP5* in serotype D, we suggest that the tryptophan biosynthetic pathway is essential for survival in both *C*. *neoformans* serotypes A and D.

### Non preferred nitrogen source improves tryptophan uptake in *trp*3.1i and *trp*5.1i mutants

The threonine biosynthetic pathway has also been considered essential in *C*. *neoformans* [27]. This condition has been attributed to the presence of few permeases or their low affinity to the substrate in *C*. *neoformans*. Also, a tight regulation of the permeases by the preferable nitrogen source (ammonium sulfate) may be the cause of low amino acid transport; however, these hypotheses have not been experimentally addressed.

**Fig 5:**
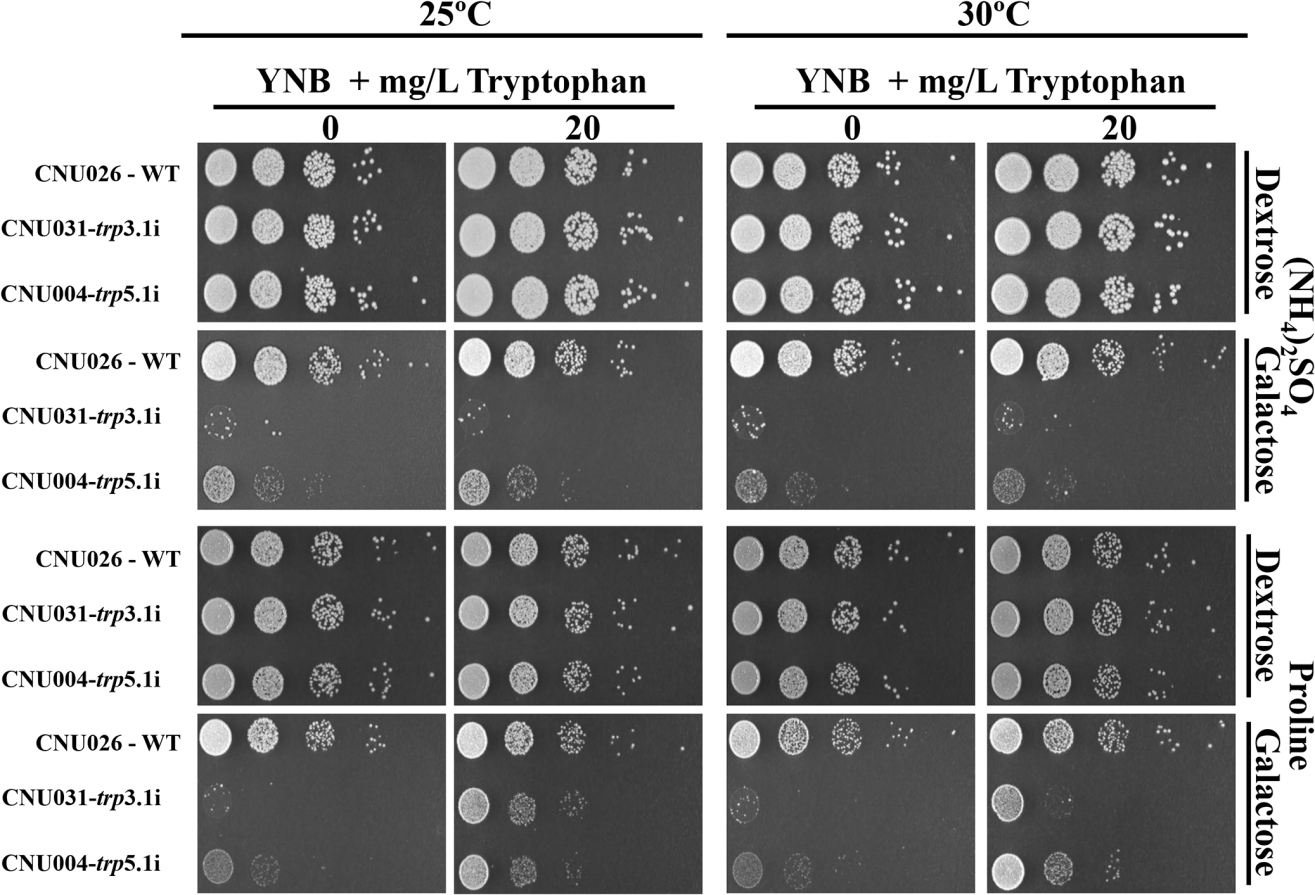
*trp3i* and *trp5i* phenotype in synthetic medium. Tryptophan uptake by *trp*3.1i (CNU031) and *trp*5.1i (CNU004) mutants was compared to the wild-type (CNU026) in synthetic medium (YNB) under preferred (NH4)_2_SO_4_ and non preferred nitrogen sources (proline) at 25º and 30ºC. Five serial dilutions (10^4^ to 1 cell) were spotted on plates containing YNB plus (NH4)_2_SO_4_ or proline, dextrose (repressing condition) or galactose (inducing condition), with and without 20 mg/L tryptophan.

The control (CNU026) and the RNAi mutants for *TRP3* and *TRP5*, CNU0031 and CNU004, respectively, were diluted and spotted on YNB without amino acids containing ammonium sulfate or proline as the sole nitrogen source (dextrose or galactose), supplemented or not with tryptophan at 25 and 30°C. Fig 5 shows that all strains grew to the same extent in dextrose regardless of nitrogen source, tryptophan supplementation or growth temperature. Regarding the *TRP3* RNAi mutant in galactose and tryptophan, the use of proline as the sole nitrogen source greatly improved growth, probably due to the more efficient intracellular transport of tryptophan. Also, the same medium supplemented with proline and tryptophan improved growth at 25°C even further. The RNAi mutant for *TRP5* was also able to uptake tryptophan in the presence of proline. However, it did so without a detectable effect on temperature.

In spite of the growth induced by possible NCR alleviation (proline as the sole nitrogen source), mutant growth was never restored to wild type levels, indicating that perturbations in the tryptophan biosynthetic pathway are deleterious, even in the presence of higher tryptophan uptake. A growth curve with increasing amounts of tryptophan supplementation (0 to 200 mg/L) was performed in order to account for the concentration effect in all nitrogen conditions tested, but no gain in growth was achieved by increasing the amount of the amino acid in the medium. This suggests that poor growth is not due to insufficient tryptophan in the growth medium.

Kingsbury & McCusker (2008) found that growth can be partially restored in the threonine mutants by the use of dipeptides. We tested Trp-Met, Trp-Ala, Trp-Trp and Trp-Pro dipeptide supplementation individually and as a pool and found no growth improvement.

The fact that proline as the sole nitrogen source improves amino acid uptake indicates that NCR play a role in permease expression; however, that may not be the only reason for essential tryptophan biosynthesis, since the mutants cannot survive in rich medium either. In this case, we would have to assume that permease expression and activity are low. Several attempts to select *trp* ^-^ mutants on YNB + proline and antibiotic (G418 or Hygromycin), a condition that should alleviate NCR and promote amino acid uptake, have failed, supporting the idea that multiple factors contribute to the essentiality of this amino acid’s biosynthetic pathway.

### Amino acid permease genes in *C*. *neoformans*

Our data revealed tryptophan as the second essential amino acid biosynthetic pathway in *C*. *neoformans* [27]. The only way an auxotroph can survive is by amino acid uptake from medium. Therefore, it is possible that essentiality may be caused by few permeases or low affinity to substrate, leading to low amino acid uptake and growth arrest. In order to address this hypothesis, we used the amino acid sequence of 24 amino acid permeases of the *APC* family present in the *S*. *cerevisiae* genome, including the high affinity ones (S2 Table), as queries to search for *C*. *neoformans* amino acid permease homologues. All 24 queries showed high similarity to 8 annotated genes, which we named *AAP*1 to *AAP*8 (S7 Table). *UGA*4, *MUP*1 and *MUP*3 (GABA, high and low affinity methionine permeases, respectively) showed sequence similarity to genes other than *AAP*1 to *AAP* 8.

*AAP*1 and *AAP*2 have 80.9% and *AAP*4 and *AAP*5 have 89.5% sequence similarity, suggesting they may be redundant. *AAP*3 is more similar to *AAP*1 and *AAP* 2 (49%). Permeases *AAP*6 and *AAP*7 have 41.1% similarity and *AAP*8 shares the least similarities with the other permeases (S1 Fig).

The transcriptional profile of all eight permeases was evaluated by Real-Time PCR in the wild type strain under different growth conditions with rich medium as the reference. Amplifications for *AAP*3 and *AAP*7 were below the threshold level; therefore, they were considered inactive in the conditions applied here and they were not analyzed further.

Comparing rich (YEPD) and synthetic dextrose medium (SD), permeases *AAP*2 to *AAP*8 display a striking increase in transcription under all types of SD mediums tested, regardless of the nitrogen source or amino acid supplementation (S2 Fig).

**Fig 6:**
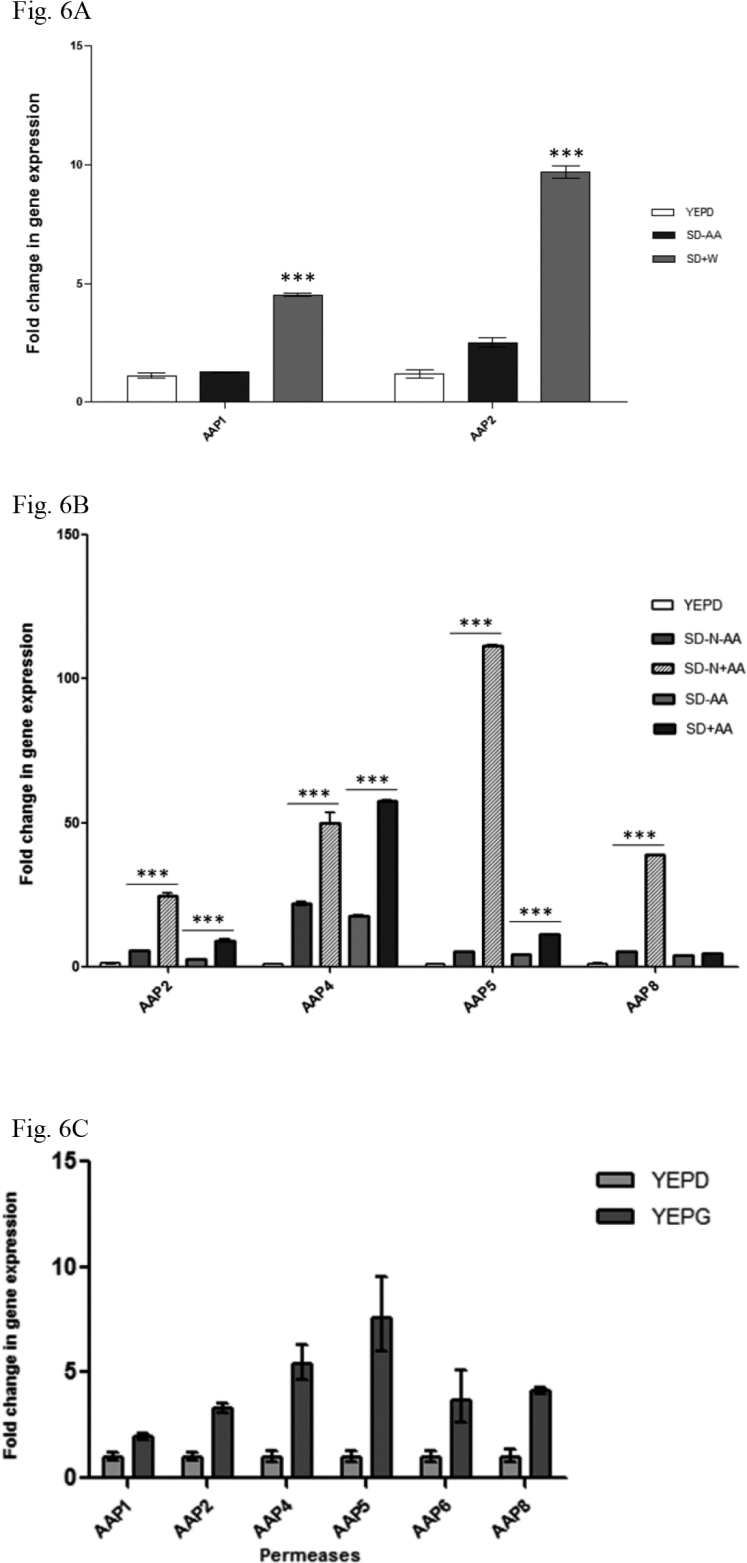
Expression profile of *C*. *neoformans* permeases by qPCR. (A) graph showing the effect of tryptophan supplementation (SD–AA vs. SD+tryptophan) on permease *AAP*1 and *AAP*2 expression; (B) shows the strong inducing effect of amino acids as the sole nitrogen source (SD-N+AA) on the expression of permeases *AAP*2, 4, 5 and 8, and the NCR effect of ammonium sulfate on permeases *AAP*2, 5 and 8 (SD-N+AA vs. SD-AA and SD+AA); (C) shows the effect of carbon catabolite repression on amino acid permeases. SD = synthetic dextrose with ammonium sulfate; SD+AA = synthetic dextrose added with ammonium sulfate and amino acids (histidine, tryptophan and methionine); SD-AA = synthetic dextrose added with ammonium sulfate without amino acids; SD+W = synthetic dextrose added with ammonium sulfate and tryptophan; SD-N+AA = synthetic dextrose without ammonium sulfate added with amino acids; SD-N-AA = synthetic dextrose without ammonium sulfate and without amino acids; YEPD = yeast extract peptone and dextrose; YEPG = yeast extract, peptone and galactose. Bars represent standard errors of 3 biological replicates. Differences are significant if *p* < 0.05 (***).

Next, we inquired if the addition of tryptophan to synthetic medium would increase the expression of *AAP* genes. As shown in Fig 6A, shifting the culture from rich (YEPD) to synthetic medium without addition of amino acids (SD-AA) resulted in 1.2 and 2.5 fold increases on *AAP*1 and *AAP*2 expression (respectively). Whereas, the addition of tryptophan to synthetic medium (SD+W) led to 4.5 and 9.6 fold increase in *AAP*1 and *AAP*2 expression, respectively. This increase represents a significant augmentation of 3.5 (*AAP*1) and 3.8 (*AAP*2) fold promoted by tryptophan addition to SD-AA (*p* < 0.05).

Also, we asked if a combination of amino acids (L-tryptophan, L-methionine and L-histidine) with or without ammonium sulfate would promote higher expression than that observed for tryptophan. Fig 6B shows that amino acids as the sole nitrogen source are capable of the highest induction of *AAP*5, followed by *AAP*8, 2 and 4 (21, 7.5, 4.2 and 3.3-fold inductions, respectively; *p* < 0.05). This result suggests that a regulatory mechanism operates in response to extracellular amino acids, leading to the transcriptional induction of these four permeases.

The addition of (NH_4_)_2_SO_4_ (preferred nitrogen source), regardless of amino acids, caused a significant reduction in the expression of *AAP*5, *AAP*8 and *AAP*2, (SD-N+AA vs. SD+N-AA and SD+N+AA), suggesting that a regulatory mechanism controlled by the preferred nitrogen source, such as nitrogen catabolism repression (NCR), may also contribute to the transcriptional regulation of these 3 permeases (Fig 6B). Clearly, *AAP*4 permease responds mainly to the presence of amino acids rather than the preferable nitrogen source, indicating that NCR may not regulate this permease.

We also inquired whether the permeases would be affected by the carbon source. The wild-type strain H99 was cultivated in rich medium (YEP) supplemented with dextrose (a preferred carbon source) or galactose (the non-preferred carbon source). Expression of all eight permeases under investigation was evaluated by qPCR. As expected, permeases *AAP*3 and *AAP*7 were amplified only below the threshold level, and therefore, were considered them inactive. The graph in Fig 6C clearly shows that galactose induced the expression of the remaining permeases, indicating they are all subject to CCR (carbon catabolite repression).

Taken together, these data suggest that: (i) *C*. *neoformans* encodes fewer permeases than *S*. *cerevisiae* (8 general permease genes and 2 methionine high and low affinity permeases); (ii) under rich medium, the permeases have the lowest transcriptional pattern observed; (iii) tryptophan can only induce permeases *AAP*1 and 2 at modest levels and, in *C*. *neoformans*, no specific high affinity tryptophan permease like *S*. *cerevisiae TAT*2 could be identified in the BLAST search; (iv) *AAP*2, 4, 5 and 8 have the highest expression when cultivated in the presence of a combination of amino acids (rather than specific ones) as the sole nitrogen source and (v) permeases *AAP*2, 5 and 8, but not *AAP*4, may be subject to nitrogen catabolism repression, since their transcription decreases under the preferable nitrogen source condition (ammonium sulfate).

### Transcriptional profile of the tryptophan biosynthetic genes

**Fig 7:**
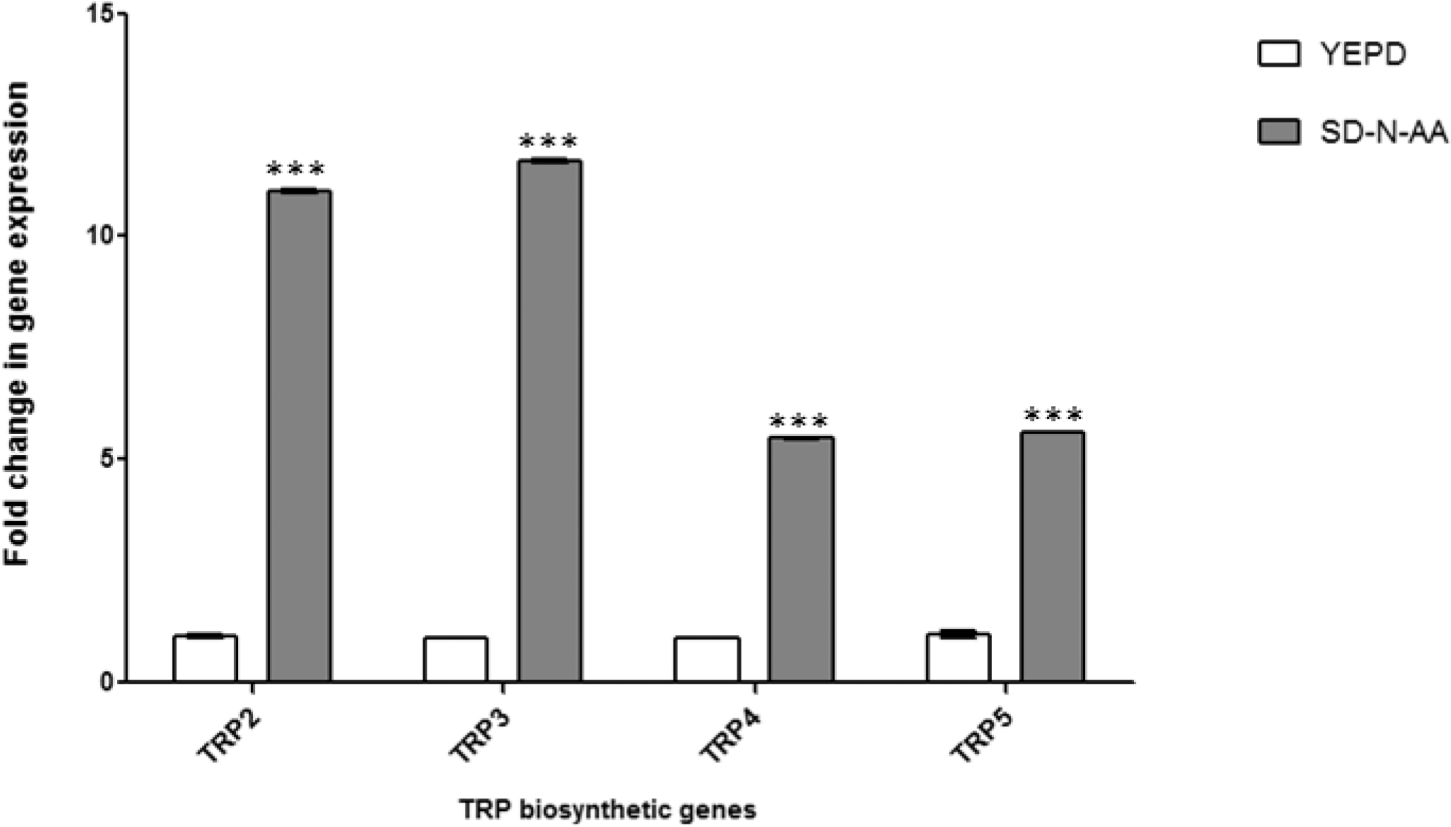
Expression profile of the tryptophan biosynthetic genes. *TRP2*, *TRP3*, *TRP4* and *TRP5* transcripts were evaluated by qPCR in response to nutritional deprivation of the nitrogen source (SD-N-AA). Bars represent standard errors of 3 biological replicates. Differences are significant if *p* < 0.05 (***).

The permease transcriptional profiling results raised the question about how the transcription of the *TRP* genes would be affected by different nutritional statuses in a *C*. *neoformans* wild-type strain. We evaluated the expression profile of the four *TRP* genes under the same conditions in which we studied the permeases. Fig 7 shows that nitrogen starvation is the condition in which *TRP* genes are fully induced. In the other conditions, only minor or no transcriptional pattern shifts were detected. This is consistent with the literature, since it is known that in *S*. *cerevisiae*, uncharged tRNA generated by nitrogen starvation triggers global amino acid control (GAAC), which leads to the induction of more than 500 genes, including several amino acid biosynthetic genes [17, 66].

### Proteins encoded by *TRP3* and *TRP5* genes are targets of inhibitors

One of the main interests behind finding an essential pathway in a pathogen is the possibility of developing a novel target for drug treatment that can be directed towards the proteins in the pathway. In this work, the initial idea was to evaluate the tryptophan biosynthetic pathway as a putative molecular target, which we accomplished, along with identifying the tryptophan biosynthetic genes *TRP3* and *TRP5* as being essential in this pathway.

The *TRP5* inhibitor N-(3-Indolylacetyl)-DL-aspartic acid acts as an allosteric effector and perturbs the TS β-active site. On the other hand, N-(3-Indolylacetyl)-L-alanine and N-(3-Indolylacetyl)-L-valine behave as competitive inhibitors of the TS α-subunit. In our experiments, we calculated the MIC for each one of these inhibitors and found that only N-(3-Indolylacetyl)-DL-aspartic acid promoted growth arrest. However, inhibition was achieved at 50 mM, which was considered physiologically irrelevant, since, at the millimolar scale, this inhibitor would be required in large amounts to promote inhibition.

Next, we wanted to test an inhibitor expected to bind and inhibit the enzyme complex antharnilate synthase. 6-diazo-5-oxo-L-norleucine (DON) inhibits glutamine amidotransferase activity in Anthranilate synthase COII, since it is a glutamine analogue [41]. Table 2 shows that DON inhibits *Cryptococcus* growth at the μMolar scale in rich medium, and interestingly, *C*. *neoformans* serotype A is the most sensitive (62,5 μM), whereas *C*. *gattii* serotype B is most resistant strain (500 μM). Considering that DON has the potential to blocks tryptophan biosynthesis and since we have shown that amino acid permease expression is repressed in rich medium and induced in synthetic dextrose medium, we asked if the MIC for DON would change under these conditions. Table 2 shows the switch from rich to synthetic medium dramatically reduced the MIC for all *Cryptococcus* species and serotypes tested. The use of preferred (ammonium sulfate) or non-preferred nitrogen source (proline) and tryptophan caused no change in the MIC, except for *C*. *neoformans* serotype D. This result indicated that all four strains have serious growth impairment when tryptophan biosynthesis is blocked, even when the best tryptophan assimilation condition is provided. Probably, since DON is a glutamine analogue widely used by the cell as a primary nitrogen source, it might block other important aspects of metabolism leading to high sensitivity. During the course of this work we provided genetic evidence that tryptophan biosynthesis is essential. Now, we showed a similar phenotype using a pharmacological tool.

**Table 2:** Minimum inhibitory concentration (in μM) of 6-diazo-5-oxo-L-norleucine (DON) determined by broth microdilution method.

Species	μM DON			
	Serotype	Rich medium	SD + NH_4_ + Trp	SD + Proline + Trp
*C*. *neoformans*	A (KN99)	62.5 (98% ± 0)	3.9 (95% ± 0)	3.9 (99% ± 0)
	D (JEC21)	125.0 (96% ± 2)	31.3 (97% ± 1)	0.9 (91% ± 5)
*C*. *gattii*	C (NIH312)	125.0 (94% ± 4)	0.78 (97% ± 5)	0.78 (99% ± 1)
	B (R265)	500.0 (90% ± 0)	1.9 (91% ± 11)	1.9 (91% ± 11)

Numbers in parenthesis represent the average percentage inhibition at the MIC and standard deviation. Trp = tryptophan.

## Discussion

The ability to cope with limiting and diverse nutritional conditions is important for microbial adaptation to various environments. Microbes are highly exposed to these changing conditions and require a plastic metabolism to survive and succeed in these situations. Yeasts are able to sense nutrients available in the surrounding environment and trigger a response that allows the uptake, biosynthesis or recycling of building blocks for cellular physiology. The choice is driven by the best resource usage and energy savings. Amino acids are among the essential components that can be acquired by uptake, biosynthesis or recycling, and *S*. *cerevisiae* contains a large amount of information about the mechanisms underlying these important metabolic processes [17, 54]. In *C*. *neoformans*, biosynthesis is one aspect of amino acid acquisition that has been studied and linked to pathogenesis in the last 10 years. Several pathways have been associated with virulence attenuation in animal models of infection, which would serve as putative molecular targets for antifungal development [27–32, 67, 68].

Notable differences in the configuration of biosynthetic genes are found among *S*. *cerevisiae* and *C*. *neoformans*. Often, different catalytic activities that are encoded by separate genes in *S*. *cerevisiae* are combined as chimeras in *C*. *neoformans* [32], giving rise to proteins that act in more than one biochemical step in a pathway. This feature was also observed in this study, since we described the *TRP3* gene encoding a likely trifunctional protein that evolved as the fusion between *TRP1* and *TRP3*. Our extended bioinformatics analysis yielded the same arrangement in two other Basidiomycetes, *U*. *maydis* and *C*. *cinerea* (data not shown).

The other aspect that is different between these two model system yeasts is the impact that the biosynthetic pathway has on the survival of *C*. *neoformans*; apparently, this opportunistic pathogen is more dependent upon biosynthesis than uptake, and this argument is based on several pieces of evidence. Kingsbury and McCusker (2008) reported that the threonine biosynthetic pathway is essential in *C*. *neoformans*. In this work, we report tryptophan as a second amino acid biosynthetic pathway that is required for *in vitro* survival. Our data showed that *trp*3i and *trp*5i mutants do not grow at RNAi inducing conditions in rich medium, where tryptophan is available in abundance (Fig 4). In agreement with Kingsbury and McCusker, we believe this fact could be explained by the low amino acid uptake caused by the few permeases encoded by the genome, the low affinity of these transporters to their substrate and tight regulation of permeases by the preferred nitrogen source. This idea was corroborated by our bioinformatics data, which showed that *C*. *neoformans* has fewer permeases and lacks a tryptophan high affinity transporter similar to *S*. *cerevisiae TAT*2. The lack of *TAT*2 may explain why even after alleviating the nitrogen catabolism repression on permeases, growth was never restored to wild type levels.

To gain insights about the role of amino acid uptake on the survival of *C*. *neoformans*, we created an inventory of amino acid permeases in *C*. *neoformans*. When we used the 24 permeases of the APC family of *S*. *cerevisiae* as query to search the *C*. *neoformans* genomes, we found eight permease genes. Among the *S*. *cerevisiae* permeases with high and low affinity to specific amino acids (*GNP*1, *LYP*1, *TAT*2, *PUT*4), only *MUP*1, *MUP*3 and *UGA*4 (high and low methionine affinity and high GABA affinity) were found to have homologues outside the group with the eight readily encountered permeases. In summary, it seems that *C*. *neoformans* has eight global affinity and two methionine permease homologues (high affinity *MUP*1 and low affinity *MUP*3), which is a considerably smaller number than the 24 *S*. *cerevisiae* permeases. We then searched the genome of other Asco- and Basidiomycetes to study their permease organization. Our results showed the very same organization in *U*. *maydis* and *C*. *cinerea*. On the other hand, *A*. *nidulans*, *C*. *albicans*, *N*. *crassa* and *S*. *pombe* all have a much more diverse and larger set of permeases (S8 Table).

The improvement in growth noted in this work (Fig 5) and reported in the literature, which is presumably caused by higher amino acid uptake under a non-preferred nitrogen source, suggests that permease genes may be under genetic regulatory mechanisms. The expression profile of these genes by qPCR indicated they have a complex regulation at the transcriptional level which is dependent upon: (i) nitrogen and carbon sources (NCR and CCR), (ii) extracellular amino acids and (iii) nutritional deprivation (Fig 6). In *S*. *cerevisiae*, the genetics underlying these 3 nutritional conditions are well-known [17, 53, 54, 56]. In *C*. *neoformans*, the effects of the preferable nitrogen source (ammonium) on the transcription of the genes necessary for assimilation of less preferable sources (such as uric acid and creatinine) have been described [20, 69]. In this work, we show the same NCR effect on permeases (*AAP*2, 5 and 8), which should impact on the efficiency of amino acid uptake. A signaling pathway such as *S*. *cerevisiae* SPS-sensing that responds to the presence of extracellular amino acids, promoting uptake, has not been described in *C*. *neoformans*. Among the main elements of this regulatory network (*SSY*1, *PTR*3, *SSY*5, *STP*1 and *STP*2), only *SSY*1 and *STP*2 seem to have homologues in *C*. *neoformans* (data not shown). There are no reports in the literature for *C*. *neoformans* regarding global amino acid control (GAAC) triggered by nitrogen starvation and uncharged tRNAs. However, homologues for several elements of this pathway can be found in the *C*. *neoformans* genome (data not shown). Nevertheless, our study suggests that this control system operates in *C*. *neoformans*, especially because our data showed that the permeases and tryptophan biosynthetic genes seem to have an expression profile driven by nitrogen deprivation, similar to global amino acid control (GAAC) in *S*. *cerevisiae*. In spite of the evidence presented here, these last two regulatory mechanisms remain to be demonstrated in *C*. *neoformans*.

In summary, we demonstrated that the tryptophan biosynthetic pathway is essential in *C*. *neoformans*. We suggest that essentiality is linked to the low number of permease genes encoded by the genome and their complex regulation, which is revealed by nutritional status. However, why gene deletions of some amino acid biosynthetic pathways are lethal and others are not is not completely clear. The methionine pathway is not essential but nonetheless deeply impacts virulence [29, 30, 68]; however, specific high and low affinity methionine permeases (*MUP*1 and *MUP*3) seem to be present in *C*. *neoformans*. Whether those permeases are specific to methionine and facilitate its uptake remains to be shown. Other pathways (isoleucine, valine and lysine) are not essential, but growth, survival and virulence are decreased in the auxotroph mutants [28, 32]. The uptake of these amino acids could be more efficient due to protein conformation and higher uptake could be just enough for mutant rescue, but still not enough to confer wild type growth, even with abundant supplementation.

Also in this work, we tested anti-metabolites and inhibitor against anthranilate phosphoribosyl transferase, anthranilate synthase and tryptophan synthase. Regarding AS we obtained remarkable inhibition (DON), at the μmolar level, showing the potential of inhibitors directed to this target. Other inhibitors have been reported in the literature and could be tested as they become available [34–40, 43, 46, 49, 70]. The next step in this investigation will be the search for novel inhibitors that have these proteins as targets. Also, the crystal structure of the enzymes should be elucidated, and inhibitors should be searched for in chemical libraries or alternatively, designed.

In conclusion, this work not only broadens the knowledge about amino acid acquisition in *C*. *neoformans*, but also indicates that the tryptophan biosynthetic pathway may be a good choice as a molecular target for novel antifungal development

## Supporting Information

S1 Fig Sequence similarity among *C*. *neoformans* permeases. AAP1 to AAP8 amino acid sequences were aligned by ClustalW in MegaAlign module of Lasergene software (DNAStar).
(DOCX)

S2 Fig Expression profile of six permease genes in *C*. *neoformans* in response to nutritional status. *AAP*1, 2, 4, 5 6, and 8 transcripts were evaluated by qPCR under rich medium (YEPD) and synthetic glucose medium (SD) plus amino acids as sole nitrogen source (SD-N+AA), without ammonium sulfate and amino acids (SD-N-AA), with tryptophan as sole nitrogen source (SD-N+W) or SD plus ammonium sulfate, minus amino acids (SD+N-AA), plus amino acids (SD+N+AA) or without tryptophan (SD+N+W).
(DOCX)

S1 Table List of strains used in this work. (DOCX)

S2 Table *S*. *cerevisiae* permease genes (1 to 24) and tryptophan biosynthetic genes (25 to 29) used as query for BLASTp search of the *C*. *neoformans* genome serotype A at Broad Institute. (DOCX)

S3 Table Primers used in this work. (DOCX)

S4 Table Deletions and selectable markers. (DOCX)

S5 Table Plasmids used and constructed in this work. (DOCX)

S6 Table Number of transformants selected after *TRP* gene deletion attempts. (DOCX)

S7 Table Permease genes denomination in *C*. *neoformans*. (DOCX)

S8 Table List of putative amino acid permeases in Asco and Basidiomycetes. (DOCX)
