# Supplementary material for: The Role of Amino Acid Permeases and Tryptophan Biosynthesis in Cryptococcus neoformans Survival
Source: PLoS One. 2015 Jul 10;10(7):e0132369. doi: 10.1371/journal.pone.0132369 (PMC4498599; doi:10.1371/journal.pone.0132369)
Supplement: S1 Fig — AAP1 to AAP8 amino acid sequences were aligned by ClustalW in MegaAlign module of Lasergene software (DNAStar). (DOCX) [file pone.0132369.s001.docx]

**S1 Fig.:** Sequence similarity among *C. neoformans* permeases. AAP1 to AAP8 amino acid sequences were aligned by ClustalW in MegaAlign module of Lasergene software (DNAStar).

Amino Acid Substitution per 100 residues

0

176.9

20

40

60

80

100

120

140

160

CNAG_07367 AAP5

CNAG_00597 AAP4

CNAG_07902 AAP2

CNAG_02539 AAP1

CNAG_01118 AAP3

CNAG_05345 AAP7

CNAG_07449 AAP6

CNAG_00574 AAP8

80,9%

89,5%

41,4%
