# Supplementary material for: The Role of Amino Acid Permeases and Tryptophan Biosynthesis in Cryptococcus neoformans Survival
Source: PLoS One. 2015 Jul 10;10(7):e0132369. doi: 10.1371/journal.pone.0132369 (PMC4498599; doi:10.1371/journal.pone.0132369)
Supplement: S2 Fig — AAP1, 2, 4, 5 6, and 8 transcripts were evaluated by qPCR under rich medium (YEPD) and synthetic glucose medium (SD) plus amino acids as sole nitrogen source (SD-N+AA), without ammonium sulfate and amino acids (SD-N-AA), with tryptophan as sole nitrogen source (SD-N+W) or SD plus ammonium sulfate, minus amino acids (SD+N-AA), plus amino acids (SD+N+AA) or without tryptophan (SD+N+W). (DOCX) [file pone.0132369.s002.docx]

**S2 Fig.:** Expression profile of six permease genes in *C. neoformans* in response to nutritional status. *AAP*1, 2, 4, 5 6, and 8 transcripts were evaluated by qPCR under rich medium (YEPD) and synthetic glucose medium (SD) plus amino acids as sole nitrogen source (SD-N+AA), without ammonium sulfate and amino acids (SD-N-AA), with tryptophan as sole nitrogen source (SD-N+W) or SD plus ammonium sulfate, minus amino acids (SD+N-AA), plus amino acids (SD+N+AA) or without tryptophan (SD+N+W).
