# Supplementary material for: The Role of Amino Acid Permeases and Tryptophan Biosynthesis in Cryptococcus neoformans Survival
Source: PLoS One. 2015 Jul 10;10(7):e0132369. doi: 10.1371/journal.pone.0132369 (PMC4498599; doi:10.1371/journal.pone.0132369)
Supplement: S1 Table — (DOCX) [file pone.0132369.s003.docx]

**S1 Table:** Strains used in this work

| **Name** | **Genotype** | **Source** |
| --- | --- | --- |
| HB101 (*E. coli*) | *F- mcrB mrr hsdS20(rB- mB-) recA13 leuB6 ara-14 proA2 lacY1 galK2 xyl-5 mtl-1 rpsL20(SmR) glnV44* λ- | Promega (Wisconsin, USA) |
| H99 (*C. neoformans serotype A*) | Wild type | Our collection |
| R265 (*C. gatti serotype B*) | Wild type | ATCC MYA-4093 |
| NIH312 (*C. gatti serotype C*) | Wild type | ATCC 34880 |
| JEC21 (*C. neoformans serotype D*) | Wild type | Our collection |
| CNU004 (*C. neoformans serotype D*) | Gal7::*TRP5i* | This work |
| CNU007 (*C. neoformans serotype D*) | pIBB without insert | This work |
| CNU026 (*C. neoformans serotype D*) | pIBB without insert | This work |
| CNU031 (*C. neoformans serotype D*) | Gal7::*TRP3i* | This work |
