# Supplementary material for: The Role of Amino Acid Permeases and Tryptophan Biosynthesis in Cryptococcus neoformans Survival
Source: PLoS One. 2015 Jul 10;10(7):e0132369. doi: 10.1371/journal.pone.0132369 (PMC4498599; doi:10.1371/journal.pone.0132369)
Supplement: S2 Table — (DOCX) [file pone.0132369.s004.docx]

**S2 Table:** *S. cerevisiae* permease genes (1 to 24) and tryptophan biosynthetic genes (25 to 29) used as query for BLASTp search of the *C. neoformans* genome serotype A at Broad Institute.

|  | **Systematic ORF** | **Gene Name** | **Substrate** | **CNAG** |
| --- | --- | --- | --- | --- |
| 1 | YBR068c | *BAP2* | Branched-chain amino acids (leucine, valine and isoleucine) | CNAG_07902.7 CNAG_00597.7 CNAG_07449.7  CNAG_02539.7 CNAG_07367.7 CNAG_00574.7  CNAG_01118.7 CNAG_05345.7 |
| 2 | YBR069c | *VAP1/TAT1/TAP1* | Valine, leucine, isoleucine, tyrosine and tryptophan | CNAG_02539.7 CNAG_00597.7  CNAG_07902.7  CNAG_01118.7 |
| 3 | YBR132c | *AGP2* | Amino acids (general) | CNAG_07449.7 CNAG_07902.7 CNAG_07367.7 CNAG_02539.7  CNAG_05345.7 CNAG_00597.7  CNAG_01118.7 |
| 4 | YCL025c | *YCC5* | Asparagine; glutamine | CNAG_02539.7 CNAG_00597.7  CNAG_07902.7 CNAG_07367.7  CNAG_01118.7 |
| 5 | YDL210w | *UGA4* | GABA (high-afinity) | CNAG_01535.7  CNAG_05017.7  CNAG_02455.7 |
| 6 | YDR046c | *PAP1* | Isoleucine, valine | CNAG_02539.7 CNAG_00597.7  CNAG_07902.7 CNAG_07367.7  CNAG_01118.7 CNAG_05345.7 |
| 7 | YDR160w | *SSY1* | Amino acid sensor system (Ssy1p-Ptr3p-Ssy5p) | CNAG_07902.7 CNAG_00597.7  CNAG_02539.7 CNAG_07367.7  CNAG_01118.7 |
| 8 | YDR508c | *GNP1* | Glutamine (high-afinity) | CNAG_02539.7 CNAG_00597.7  CNAG_07902.7 CNAG_05345.7  CNAG_01118.7 CNAG_07367.7 |
| 9 | YEL063c | *CAN1* | Arginine, lysine, ornithine and canavanine | CNAG_07902.7 CNAG_00597.7 CNAG_05345.7  CNAG_02539.7 CNAG_07367.7  CNAG_01118.7 CNAG_07449.7 |
| 10 | YFL055w | *AGP3* | Low-affinity amino acid permease (general) | CNAG_02539.7 CNAG_00597.7  CNAG_07902.7 CNAG_07367.7  CNAG_01118.7 CNAG_05345.7 |
| 11 | YGL077c | *HNM1/CTR1* | Choline | CNAG_02455.7  CNAG_05119.7 |
| 12 | YGR055w | *MUP1* | Methionine (high-afinity) | CNAG_07693.7 CNAG_03955.7 |
| 13 | YGR191w | *HIP1* | Histidine | CNAG_07902.7 CNAG_07367.7  CNAG_02539.7 CNAG_00597.7  CNAG_01118.7 CNAG_05345.7 |
| 14 | YHL036w | *MUP3* | Methionine (low-afinity) | CNAG_07693.7 CNAG_03955.7 |
| 15 | YKL174c | YKL174c | Choline | CNAG_05119.7 CNAG_05017.7 |
| 16 | YKR039w | *GAP1* | Amino acids (general) [naturally occurring L-amino acid, Q-aminobutyrate, ornithine, citrulline, some D-amino acids and some toxic analogues | CNAG_02539.7  CNAG_07902.7  CNAG_01118.7  CNAG_00597.7  CNAG_07367.7  CNAG_05345.7  CNAG_07449.7 |
| 17 | YLL061w | *MMP1* | S-MethylMethionine Permease | CNAG_07902.7  CNAG_02539.7  CNAG_00597.7 |
| 18 | YNL268w | *LYP1* | Lysine (high-afinity) | CNAG_07902.7 CNAG_00597.7 CNAG_05345.7  CNAG_02539.7 CNAG_07367.7  CNAG_01118.7 CNAG_07449.7 |
| 19 | YNL270c | *APL1/ALP1* | Basic amino acids  Arginine transporter | CNAG_07902.7 CNAG_00597.7 CNAG_05345.7  CNAG_02539.7 CNAG_07367.7  CNAG_01118.7 CNAG_07449.7 |
| 20 | YNR056c | *BIO5* | Involved in biotin synthesis | CNAG_02455.7 CNAG_05017.7  CNAG_01535.7 CNAG_00574.7  CNAG_05119.7 |
| 21 | YOL020w | *TAT2/SCM2/ TAP2/LTG3* | Tryptophan (high-afinity) | CNAG_07902.7 CNAG_00597.7 CNAG_07449.7  CNAG_02539.7 CNAG_07367.7  CNAG_01118.7 CNAG_05345.7 |
| 22 | YOR348c | *PUT4* | Proline and Q-aminobutyrate (high-afinity) | CNAG_07902.7 CNAG_00597.7 CNAG_07449.7  CNAG_02539.7 CNAG_07367.7  CNAG_01118.7 CNAG_05345.7 |
| 23 | YPL265w | *DIP5* | Dicarboxylic amino acids | CNAG_00597.7 CNAG_07902.7 CNAG_07449.7  CNAG_07367.7 CNAG_05345.7  CNAG_02539.7 CNAG_01118.7 |
| 24 | YPL274w | *SAM3* | S-adenosylmethionine (high-affinity) | CNAG_02539.7  CNAG_07902.7  CNAG_00597.7  CNAG_01118.7  CNAG_07367.7 |
| 25 | YDR007W | *TRP1* | ASCO II | CNAG_04501.2 |
| 26 | YER090W | *TRP2* | ASCO I | CNAG_06679.2 |
| 27 | YKL211C | *TRP3* | ASCO II | CNAG_04501.2 |
| 28 | YDR354W | *TRP4* | Anthranilate phosphoribosyl transferase | CNAG_00811.2 |
| 29 | YGL026C | *TRP5* | Tryptophan synthase | CNAG_00649.2 |
