# Supplementary material for: The Role of Amino Acid Permeases and Tryptophan Biosynthesis in Cryptococcus neoformans Survival
Source: PLoS One. 2015 Jul 10;10(7):e0132369. doi: 10.1371/journal.pone.0132369 (PMC4498599; doi:10.1371/journal.pone.0132369)
Supplement: S3 Table — (DOCX) [file pone.0132369.s005.docx]

**S3 Table:** List of primers used in this work.

| **Name** | **Sequence (5´to 3´)** | **Use** |
| --- | --- | --- |
| PRCP100 | GTTTCTAAGTCTACGTATACATGA | *TRP2* H99 Deletion Cassete Construction |
| PRCP101 | CTCCAGCTCACATCCTCGCATTTGATTTTTCCTCGGCTGTACCGA | *TRP2* H99 Deletion Cassete Construction |
| PRCP102 | TCGGTACAGCCGAGGAAAAATCAAATGCGAGGATGTGAGCTGGAG | *TRP2* H99 Deletion Cassete Construction |
| PRCP103 | ATATACACCCTCTAAGGAAAAGAAGAGATGTAGAAACTAGCTTCC | *TRP2* H99 Deletion Cassete Construction |
| PRCP104 | GGAAGCTAGTTTCTACATCTCTTCTTTTCCTTAGAGGGTGTATAT | *TRP2* H99 Deletion Cassete Construction |
| PRCP105 | TAACACACATCATCACGCTCTTGC | *TRP2* H99 Deletion Cassete Construction |
| PRCP106 | ATTAGGGCAAGATGTTTTGGG | *TRP2* H99 Deletion Cassete Construction Confirmation |
| PRCP107 | GAACACCGCAGCTCCCACTCC | *TRP2* H99 Deletion Cassete Construction Confirmation |
| PRCP108 | GGGGGGCGCGTCGTTGTCTGCGAGG | *TRP5* H99 Deletion Cassete Construction |
| PRCP109 | TCTCCAGCTCACATCCTCGCAGAAGGAGAGATGAGATGGGATACAAAC | *TRP5* H99 Deletion Cassete Construction |
| PRCP110 | GTTTGTATCCCATCTCATCTCTCCTTCTGCGAGGATGTGAGCTGGAGA | *TRP5* H99 Deletion Cassete Construction |
| PRCP111 | CTGCACATGCATATTCGATGCCTGAAGAGATGTAGAAACTAGCTTCC | *TRP5* H99 Deletion Cassete Construction |
| PRCP112 | GGAAGCTAGTTTCTACATCTCTTCAGGCATCGAATATGCATGTGCAG | *TRP5* H99 Deletion Cassete Construction |
| PRCP113 | CCAGCCCTCACGTTCTCCCCCTTC | *TRP5* H99 Deletion Cassete Construction |
| PRCP114 | GAAGAGACTGCCGGATGCG | *TRP5* H99 Deletion Cassete Construction Confirmation |
| PRCP115 | CCACAGTCCCACACCCCTC | *TRP5* H99 Deletion Cassete Construction Confirmation |
| PRCP116 | TTACTGGTAAAGACGCTCTC | *TRP4* H99 Deletion Cassete Construction |
| PRCP117 | CTCCAGCTCACATCCTCGCAAGTGGATTTTCGATGTTCGAGTGAT | *TRP4* H99 Deletion Cassete Construction |
| PRCP118 | ATCACTCGAACATCGAAAATCCACTTGCGAGGATGTGAGCTGGAG | *TRP4* H99 Deletion Cassete Construction |
| PRCP119 | CTTTTTCCTAGAGATACAGCTTTGAAGAGATGTAGAAACTAGCTTCC | *TRP4* H99 Deletion Cassete Construction |
| PRCP120 | GGAAGCTAGTTTCTACATCTCTTCAAAGCTGTATCTCTAGGAAAAAG | *TRP4* H99 Deletion Cassete Construction |
| PRCP121 | GCCATTCAGCTGTTCTTTTCTGAG | *TRP4* H99 Deletion Cassete Construction |
| PRCP122 | TAACAAGGAAAAAGTGGGCAG | *TRP4* H99 Deletion Cassete Construction Confirmation |
| PRCP123 | TTTAAAGTTATTTTGAGGACG | *TRP4* H99 Deletion Cassete Construction Confirmation |
| PRCP124 | CGGTGAATTTAAAAGATGGC | *TRP2* H99 Deletion Cassete Construction |
| PRCP125 | CTCCAGCTCACATCCTCGCATTTTGCCAGTTTGTAGCTGTATAGATG | *TRP2* H99 Deletion Cassete Construction |
| PRCP126 | CATCTATACAGCTACAAACTGGCAAAATGCGAGGATGTGAGCTGGAG | *TRP2* H99 Deletion Cassete Construction |
| PRCP127 | GCTTCTATATTGTCCAAAATCTTGAAGAGATGTAGAAACTAGCTTCC | *TRP2* H99 Deletion Cassete Construction |
| PRCP128 | GGAAGCTAGTTTCTACATCTCTTCAAGATTTTGGACAATATAGAAGC | *TRP2* H99 Deletion Cassete Construction |
| PRCP129 | AATTTCGACATTGGGGGAGG | *TRP2* H99 Deletion Cassete Construction |
| PRCP130 | TTCCGGCGTTTTCTGAAGCC | *TRP2* H99 Deletion Cassete Construction Confirmation |
| PRCP131 | CCCTCGTTTACGGTCAGAAGG | *TRP2* H99 Deletion Cassete Construction Confirmation |
| PRCP132 | CAAGGGTTGGGATGGCTACC | *TRP4* H99 Deletion Cassete Construction |
| PRCP133 | GATGGTGGTGACTTCGATGCC | *TRP4* H99 Deletion Cassete Construction |
| PRCP134 | TTGTTGAGGAATGCGGACATG | *TRP5* H99 Deletion Cassete Construction |
| PRCP135 | CCATTCTTTTCAACTTTCACC | *TRP5* H99 Deletion Cassete Construction |
| PRCP137 | CTCCAGCTCACATCCTCGCATGTGCTGGATGTGGATGAGGAGAGACGA | *TRP5* JEC21 Deletion Cassete Construction |
| PRCP138 | TCGTCTCTCCTCATCCACATCCAGCACATGCGAGGATGTGAGCTGGAG | *TRP5* JEC21 Deletion Cassete Construction |
| PRCP139 | GATAGCGTATACTACATGCAGCTGAAGAGATGTAGAAACTAGCTTCC | *TRP5* JEC21 Deletion Cassete Construction |
| PRCP140 | GGAAGCTAGTTTCTACATCTCTTCAGCTGCATGTAGTATACGCTATC | *TRP5* JEC21 Deletion Cassete Construction |
| PRCP143 | gctacacatatgGGTGTCTCTAGGGACGTGATCG | RNAi *TRP3* JEC21, NdeI |
| PRCP144 | gctacaagatctGGTGTCTCTAGGGACGTGATCG | RNAi *TRP3* JEC21, BglII |
| PRCP145 | gctacacctaggGGAAGCGACTGAATTTGGGCGG | RNAi *TRP3* JEC21, AvrII |
| PRCP146 | gctacactcgagGGAAGCGACTGAATTTGGGCGG | RNAi *TRP3* JEC21, XhoI |
| PRCP147 | gctacaactagtGGTGTCTCTAGGGACGTGATCG | RNAi *TRP3* JEC21, SpeI |
| PRCP148 | gctacaactagtGGAAGCGACTGAATTTGGGCGG | RNAi *TRP3* JEC21, SpeI |
| PRCP149 | gctacacatatgAGGATCCGAGTGTAAGACTGG | RNAi *TRP5* JEC21, NdeI |
| PRCP150 | gctacaagatctAGGATCCGAGTGTAAGACTGG | RNAi *TRP5* JEC21, BglII |
| PRCP151 | gctacacctaggTAGTCGAGACCGGCAGAGATG | RNAi *TRP5* JEC21, AvrII |
| PRCP152 | gctacactcgagTAGTCGAGACCGGCAGAGATG | RNAi *TRP5* JEC21, XhoI |
| PRCP153 | gctacaactagtAGGATCCGAGTGTAAGACTGG | RNAi *TRP5* JEC21, SpeI |
| PRCP154 | gctacaactagtTAGTCGAGACCGGCAGAGATG | RNAi *TRP5* JEC21, SpeI |
| PRCP155 | gctacaTAAACTCCCTTCTCGATTCGG | RNAi 2 *TRP5* JEC21, SpeI |
| PRCP156 | gctacaactagtATGCCAAACTTGGCGCAGAC | RNAi 2 *TRP5* JEC21, SpeI |
| PRCP157 | AATCAAAGGAAGGCCAGCTCAC | *TRP5* H99 Real-Time PCR |
| PRCP158 | CACTCAAACAGATGACGATATCC | *TRP5* H99 Real-Time PCR |
| PRCP159 | CGGTGGAGAGATCGCTTATGC | *TRP3* H99 Real-Time PCR |
| PRCP160 | CCTTCACTCATGCAACTCTCAG | *TRP3* H99 Real-Time PCR |
| PRCP161 | gctacaactagtTGCTTCTCCCTCCAAGGGC | RNAi 2 JEC21 *TRP3* SpeI |
| PRCP162 | gctacaactagtCGGTTGTTAACACCAATGACC | RNAi 2 JEC21 *TRP3* SpeI |
| PRCP165 | AGTATGACTCCACACATGGTCG | *GPDH* JEC21 Real-Time PCR |
| PRCP166 | AGACAAACATAGGAGCATCAGC | *GPDH* JEC21 Real-Time PCR |
| PRCP185 | GCCTTATGGTATACTCTATGATG | *AAP1* H99 Real-Time PCR |
| PRCP186 | CCGTATGCCCGAGCACCGAGG | *AAP1* H99 Real-Time PCR |
| PRCP187 | CATGGTATACGCGATGATGG | *AAP2* H99 Real-Time PCR |
| PRCP188 | TCTGGCTCCCCAGAAGTTAATG | *AAP2* H99 Real-Time PCR |
| PRCP189 | TCTAACCATTCTTGGTATCG | *AAP3* H99 Real-Time PCR |
| PRCP190 | ATGTACCACCGAGATAAAAG | *AAP3* H99 Real-Time PCR |
| PRCP191 | CGAGGCAAAGAACCCACG | *AAP4* H99 Real-Time PCR |
| PRCP192 | AATCAAGATGCAAGCGTTTATG | *AAP4* H99 Real-Time PCR |
| PRCP193 | ACTTACTTGGACCTCTATCCTC | *AAP5* H99 Real-Time PCR |
| PRCP194 | TTTTCGGATCAGCTTGAAACC | *AAP5* H99 Real-Time PCR |
| PRCP195 | CCTTGAAAGACCGTTTCGGC | *AAP6* H99 Real-Time PCR |
| PRCP196 | TGTCACAAGTGTTGGGTCATTG | *AAP6* H99 Real-Time PCR |
| PRCP197 | TTACATCATTTCTGCTGTGTTC | *AAP7* H99 Real-Time PCR |
| PRCP198 | CATGTATGTGAAAGCGATGG | *AAP7* H99 Real-Time PCR |
| PRCP199 | TCTCTTTCTAGGGATTCTTATC | *AAP8* H99 Real-Time PCR |
| PRCP200 | CTCCGCCATATGGGCAGAAGC | *AAP8* H99 Real-Time PCR |
| PRCP215 | AGACCGAGGAGGAGGACGCTG | *TRP2* H99 Real-Time PCR |
| PRCP216 | AAGCGTCGAATCTAGATTGGTC | *TRP2* H99 Real-Time PCR |
| PRCP217 | AGGAGATCGAGGGAATGTTCG | *TRP3* H99 Real-Time PCR |
| PRCP218 | CGTAAGCGATCTCTCCACCG | *TRP3* H99 Real-Time PCR |
| PRCP219 | GCCCAGGCCGGTGCTTTTC | *TRP4* H99 Real-Time PCR |
| PRCP220 | GGACCCATGCTTGGCGACC | *TRP4* H99 Real-Time PCR |
| PRCP221 | ATGTATCCCATCCATCGCCAC | *TRP4* H99 Real-Time PCR |
| PRCP222 | CTTGTTCATGGGCTACTACAAC | *TRP5* H99 Real-Time PCR |
| PRCP223 | ACGCCCATCTTGGAGACGAC | *TRP5* H99 Real-Time PCR |
| PRCP224 | TACTCTCCTTATCGACAACTATG | *TRP3* JEC21 Real-Time PCR |
| PRCP225 | GAAAACATCCCCTCGATCTCC | *TRP3* JEC21 Real-Time PCR |
| PRCP226 | GGAGATCGAGGGGATGTTTTC | *TRP3* JEC21 Real-Time PCR |
| PRCP227 | AAGCGATCTCTCCACCGAGC | *TRP3* JEC21 Real-Time PCR |
