# Supplementary material for: The Role of Amino Acid Permeases and Tryptophan Biosynthesis in Cryptococcus neoformans Survival
Source: PLoS One. 2015 Jul 10;10(7):e0132369. doi: 10.1371/journal.pone.0132369 (PMC4498599; doi:10.1371/journal.pone.0132369)
Supplement: S4 Table — (DOCX) [file pone.0132369.s006.docx]

**S4 table:** Deletions and selectable markers

| **Deletion type** | **Selectable markers** | **Source** |
| --- | --- | --- |
| *trp2*Δ::Neo^R^, H99 | Neomicin (Neo^R^) | This Work |
| *trp3*Δ::Hph^R^, H99 | Hygromycin B (Hph^R^) | This Work |
| *trp4*Δ::Neo^R^, H99 | Neomicin (Neo^R^) | This Work |
| *trp5*Δ::Hph^R^, H99 | Hygromycin B (Hph^R^) | This Work |
| *trp5*Δ::Neo^R^, JEC21 | Neomicin (Neo^R^) | This Work |
| *ura*4Δ::Neo^R^ | Neomicin (Neo^R^) | De Gontijo et al., 2014 |
