# Supplementary material for: The Role of Amino Acid Permeases and Tryptophan Biosynthesis in Cryptococcus neoformans Survival
Source: PLoS One. 2015 Jul 10;10(7):e0132369. doi: 10.1371/journal.pone.0132369 (PMC4498599; doi:10.1371/journal.pone.0132369)
Supplement: S5 Table — (DOCX) [file pone.0132369.s007.docx]

**S5 table:** Plasmids used and constructed in this work.

| **Name** | **Insert** | **Use** | **Source** |
| --- | --- | --- | --- |
| pGEM-T | - | Cloning | Promega (Wisconsin) |
| pIBB103 | No insert | RNAi | Skowyra & Doering, 2012 |
| pLK25 | Neo^R^/Amp^R^ | Geneticin resistance | Gift from Dr. Lukas Kozubowski |
| pZPHyg | *Hph*^R^/Amp^R^ | Hygromycin B resistance | Fraser et al., 2003 |
| pRP012 | pIBB103::*trp3.1i*, Amp^R^ | RNAi in *TRP3* | This work |
| pRP013 | pIBB103::*trp3.1i*, Amp^R^ | RNAi in *TRP3* | This work |
| pRP014 | pIBB103::*trp5.1i*, Amp^R^ | RNAi in *TRP5* | This work |
| pRP015 | pIBB103::*trp5.1i*, Amp^R^ | RNAi in *TRP5* | This work |
| pRP016 | pIBB103::*trp5.1i*, Amp^R^ | RNAi in *TRP5* | This work |
| pRP018 | pIBB103::*trp3.2i*, Amp^R^ | RNAi in *TRP3* | This work |
| pRP019 | pIBB103::*trp3.2i*, Amp^R^ | RNAi in *TRP3* | This work |
| pRP032 | pIBB103::*trp5.2i*, Amp^R^ | RNAi in *TRP5* | This work |
| pRP033 | pIBB103::*trp5.2i*, Amp^R^ | RNAi in *TRP5* | This work |
| pRP034 | pIBB103::*trp5.2i*, Amp^R^ | RNAi in *TRP5* | This work |
| pRP035 | pIBB103::*trp5.2i*, Amp^R^ | RNAi in *TRP5* | This work |
