# Supplementary material for: The Role of Amino Acid Permeases and Tryptophan Biosynthesis in Cryptococcus neoformans Survival
Source: PLoS One. 2015 Jul 10;10(7):e0132369. doi: 10.1371/journal.pone.0132369 (PMC4498599; doi:10.1371/journal.pone.0132369)
Supplement: S6 Table — (DOCX) [file pone.0132369.s008.docx]

S6 Table: Number of transformants selected after *TRP* gene deletion attempts.

| **Gene** | **YEPD + G418/YEPD + Hygromycin** | **YNB + 5-FAA** | **Auxotrophs** | **Total** |
| --- | --- | --- | --- | --- |
| *TRP*2 | 373 | 183 | 0 | 556 |
| *TRP*3 | 656 | 169 | 0 | 825 |
| *TRP*4 | 400 | 120 | 0 | 520 |
| *TRP*5 | 376 | 99 | 0 | 497 |
| Total | 1805 | 571 | 0 | 2376 |
