# Supplementary material for: The Role of Amino Acid Permeases and Tryptophan Biosynthesis in Cryptococcus neoformans Survival
Source: PLoS One. 2015 Jul 10;10(7):e0132369. doi: 10.1371/journal.pone.0132369 (PMC4498599; doi:10.1371/journal.pone.0132369)
Supplement: S7 Table — (DOCX) [file pone.0132369.s009.docx]

**S6 table:** Permease genes denomination in *C. neoformans*.

| Locus | Gene name |
| --- | --- |
| CNAG_07902.7 | *AAP2* |
| CNAG_02539.7 | *AAP1* |
| CNAG_01118.7 | *AAP3* |
| CNAG_00597.7 | *AAP4* |
| CNAG_07367.7 | *AAP5* |
| CNAG_05345.7 | *AAP7* |
| CNAG_07449.7 | *AAP6* |
| CNAG_00574.7 | *AAP8* |
