# Supplementary material for: The Role of Amino Acid Permeases and Tryptophan Biosynthesis in Cryptococcus neoformans Survival
Source: PLoS One. 2015 Jul 10;10(7):e0132369. doi: 10.1371/journal.pone.0132369 (PMC4498599; doi:10.1371/journal.pone.0132369)
Supplement: S8 Table — (DOCX) [file pone.0132369.s010.docx]

**S7 table:** List of putative amino acid permeases in Asco and Basidiomycetes.

| **Fungi** | **Loci** | | | |
| --- | --- | --- | --- | --- |
| ***U. maydis* (9)** | UM00343.1  UM01762.1  UM03325.1 UM03691.1 UM03700.1 UM04186.1 | UM05035.1 UM05269.1 UM06012.1 |  |  |
| ***C. cinerea* (8)** | CC1G_01034.3 CC1G_03688.3 CC1G_04288.3 CC1G_07574.3 CC1G_07764.3 CC1G_08094.3 CC1G_09113.3 CC1G_09573.3 |  |  |  |
| ***S. pombe* (14)** | SOCG_00140.1 SOCG_00999.1 SOCG_01114.1 SOCG_01716.1 SOCG_01859.1 SOCG_01888.1 SOCG_01896.1 SOCG_02613.1 SOCG_02630.1 SOCG_02633.1 | SOCG_02817.1 SOCG_02820.1 SOCG_02891.1 SOCG_03136.1 |  |  |
| ***N. crassa* (17)** | NCU00648.7 NCU00721.7 NCU02195.7 NCU02783.7 NCU03509.7 NCU04435.7 NCU04468.7 NCU04942.7 NCU05168.7 NCU05198.7 | NCU05576.7 NCU05830.7 NCU07129.7 NCU07175.7 NCU07754.7 NCU10276.7 NCU10675.7 |  |  |
| ***A. nidulans* (38)** | ANID_00856.1 ANID_01061.1 ANID_01581.1 ANID_01631.1 ANID_01659.1 ANID_01732.1 ANID_02201.1 ANID_02074.1 ANID_02287.1 ANID_02468.1 | ANID_02560.1 ANID_02781.1 ANID_02962.1 ANID_03081.1 ANID_03287.1 ANID_03345.1 ANID_03359.1 ANID_04328.1 ANID_04649.1 ANID_05275.1 | ANID_05678.1 ANID_05968.1 ANID_06118.1 ANID_07150.1 ANID_07242.1 ANID_07243.1 ANID_07392.1 ANID_07916.1 ANID_08279.1 ANID_08726.1 | ANID_08816.1 ANID_08990.1 ANID_09174.1 ANID_09280.1 ANID_09441.1 ANID_09490.1 ANID_10905.1 ANID_10972.1 |
| ***C. albicans* (31)** | CAWG_00136.1 CAWG_00254.1 CAWG_00332.1 CAWG_00353.1 CAWG_00703.1 CAWG_00807.1 CAWG_00834.1 CAWG_00890.1 CAWG_01125.1 CAWG_01126.1 | CAWG_01128.1 CAWG_01547.1 CAWG_02255.1 CAWG_02256.1 CAWG_02714.1 CAWG_02671.1 CAWG_02868.1 CAWG_03332.1 CAWG_03671.1 CAWG_03835.1 | CAWG_03896.1 CAWG_04047.1 CAWG_04053.1 CAWG_04223.1 CAWG_04565.1 CAWG_04662.1 CAWG_05254.1 CAWG_05263.1 CAWG_05276.1  CAWG_05325.1 | CAWG_05419.1 |
